# Complete Mitochondrial Genome of *Phoxinus grumi* (Cypriniformes: Leuciscidae): Characterization and Phylogenetic Position

**DOI:** 10.3390/genes17060635

**Published:** 2026-05-30

**Authors:** Hongxiong Chang, Wei Guo, Ping Yang, Xinyang Li, Rui Han, Jiangyuan Liu, Jia Wang

**Affiliations:** Xinjiang Key Laboratory for Ecological Adaptation and Evolution of Extreme Environment Organisms, College of Life Sciences, Xinjiang Agricultural University, Urumqi 830052, China; hongxiong1819@163.com (H.C.); guowei612@xjau.edu.cn (W.G.); zkayp1314@163.com (P.Y.); 15599773075@163.com (X.L.); lip546686@163.com (R.H.); 15736427556@163.com (J.L.)

**Keywords:** *Phoxinus grumi*, *Rhynchocypris*, Pseudaspininae, Leuciscidae, mitochondrial genome, phylogenetic relationship

## Abstract

Background: *Phoxinus grumi*, a small leuciscid fish endemic to the Turpan Basin in Xinjiang, China, has long been the subject of taxonomic disputes, hindering accurate species identification and the understanding of its evolutionary history. Methods: To resolve this uncertainty, we sequenced and characterized the complete mitochondrial genome of *P. grumi* using next-generation sequencing. Results: The circular mitogenome is 16,604 bp long and comprises the typical 13 protein-coding genes, 22 tRNA genes, two rRNA genes, and one control region, exhibiting gene overlap and intergenic spacing. The overall base composition shows a pronounced AT bias. Notably, all tRNA genes except tRNA-Ser1 fold into a typical cloverleaf secondary structure; *tRNA-Ser1* lacks the dihydrouracil (DHU) arm, representing an unusual structural variation. All 13 PCGs have been subject to purifying selection (*Ka/Ks* < 1), with *ATP8* evolving fastest and *COX1* being the most conserved. Maximum likelihood (ML) and Bayesian inference (BI) phylogenetic analyses were conducted based on the concatenated sequences of the 13 mitochondrial PCGs, as well as the *Cytb* gene. Both datasets consistently placed *P. grumi* within the subfamily Pseudaspininae, forming a strongly supported sister relationship with the genus *Rhynchocypris*. This inference was further supported by Kimura two-parameter (K2P) genetic distance analyses, which revealed the smallest divergence between *P. grumi* and *Rhynchocypris* species (0.1620–0.1703), markedly smaller than that observed between *P. grumi* and core *Phoxinus* species (0.2475–0.2558). Conclusions: Together, these results support the placement of *P. grumi* within the East Asian Pseudaspininae lineage and help clarify its taxonomic position, which has long been debated. The complete mitochondrial genome of *P. grumi* provides additional mitogenomic data for phylogenetic analyses of Leuciscidae and contributes to a better understanding of the evolutionary relationships and diversification of Far Eastern leuciscids. These findings may also provide a molecular basis for future studies on the conservation genetics and environmental adaptation of this endemic cold-water fish from the arid Turpan Basin in northwestern China.

## 1. Introduction

As the most species-rich group of vertebrates, fish are central to evolutionary biology, systematics, and resource conservation research. Clarifying their phylogenetic relationships is foundational for understanding their evolution [1]. The family Leuciscidae, belonging to the order Cypriniformes, is an important group of freshwater fishes widely distributed across Eurasia and North America, comprising multiple subfamilies and genus-level taxonomic units. The diversification and geographic distribution patterns of this family have been strongly shaped by geological evolution and environmental changes, making it an ideal model for investigating adaptive evolution and biogeographic patterns in fishes [2,3]. Most leuciscids are small fishes that typically inhabit cold, well-oxygenated environments such as mountain streams and upper river reaches, and they play important ecological roles in freshwater ecosystems [4]. In recent years, with the rapid advancement of molecular systematics, the taxonomic framework of Leuciscidae has been continually revised [3,5]. However, the phylogenetic positions of some lineages remain controversial, and convergent evolution of traditional morphological traits has blurred their taxonomic boundaries. Therefore, re-evaluating the phylogenetic relationships of leuciscid fishes using molecular systematic approaches has become a major focus of current research [6,7,8,9].

As a circular double-stranded DNA molecule, the mitochondrial genome exhibits distinct advantages, including its simple structure, high copy number, moderate evolutionary rate, maternal inheritance, and rare recombination. Consequently, it has become an important molecular marker for phylogenetic reconstruction, species identification, population genetic structure analysis, and adaptive evolution studies in fishes [10,11,12]. The fish mitochondrial genome typically contains 13 protein-coding genes, 22 transfer RNA genes, two ribosomal RNA genes, and a single non-coding control region. Features such as gene order, base composition, codon usage bias, and gene rearrangements not only reflect the genetic diversity of species but also harbor abundant evolutionary information, providing critical clues for resolving phylogenetic relationships and evolutionary histories among lineages [13,14]. With the development of high-throughput sequencing technologies, an increasing number of complete fish mitochondrial genomes have been sequenced and applied in phylogenetic research, offering important evidence for resolving controversial issues in traditional morphological taxonomy [15].

*P. grumi*, a member of Leuciscidae, is a small cold-water fish endemic to China, where it is found exclusively in streams and ditches in the Turpan Basin of Xinjiang [16,17]. Its strong adaptability to environmental factors, such as water temperature and dissolved oxygen, makes it a valuable species for studying the adaptive evolution of fish in extreme environments. As an endemic species native to the Turpan Basin, *P. grumi* plays an important role in maintaining the balance of local freshwater ecosystems. However, its narrow distribution range, water shrinkage, and anthropogenic disturbances have caused its population size to continually decline, and natural populations have become scarce in some water bodies, leading to its listing as a regionally protected species [18,19]. To date, research on *P. grumi* remains extremely limited, with most reports focusing on morphological descriptions and distribution records [17,20]. Although a chromosome-level nuclear genome assembly of *P. grumi* has recently been reported as a genomic resource [21], the mitogenomic architecture and evolutionary characteristics of *P. grumi* remain poorly understood, and comprehensive analyses of mitochondrial genome evolution, selective constraints, codon usage bias, and phylogenetic relationships are still lacking. Studying mitochondrial genomes provides complementary evolutionary information that is particularly informative for phylogenomics, lineage divergence, and molecular systematics.

As a result of increasing phylogenetic research and the continuous advancement of molecular systematics, the genus *Phoxinus* is no longer regarded as a Holarctic Cyprinoidei group (from the previously broad concept of Cyprinidae), and related North American species are no longer assigned to this genus, leading to revisions of the traditional generic taxonomy [22]. Some studies indicate that most Asian species traditionally classified as *Phoxinus* are phylogenetically more closely related to *Rhynchocypris*, and researchers have suggested that they be reclassified into this genus, with only some Asian populations of *Phoxinus phoxinus* remaining in *Phoxinus* [23,24]. *P. grumi*, with a relatively limited distribution range, is highly morphologically similar to species in the genera *Phoxinus* and *Rhynchocypris* [10,25]. Its phylogenetic position is difficult to accurately determine using traditional morphological approaches, and its taxonomic status has long been controversial [26,27,28,29], highlighting the need to analyze the complete mitochondrial genome of *P. grumi*.

To date, phylogenetic studies of Leuciscidae have mainly focused on some common genera and species [3,30], with insufficient attention paid to endemic and rare species such as *P. grumi*. This has resulted in an incomplete overall phylogenetic framework for the family and unclear divergence relationships between subfamilies and genus-level groups. In particular, the taxonomic boundaries and evolutionary relationships between the genera *Phoxinus* and *Rhynchocypris* remain highly controversial. Therefore, further revisions of the taxonomic status of relevant lineages and elucidation of their evolutionary histories urgently require more systematic molecular evidence.

Therefore, aiming to fill the above research gap, we assembled and annotated the complete mitochondrial genome of *P. grumi* using high-throughput sequencing technology, systematically analyzing its sequence to determine its genomic structural characteristics, nucleotide composition, gene order, codon usage bias, and control region structure. Phylogenetic trees were constructed using Bayesian inference (BI) and maximum likelihood (ML) methods, together with published data on the complete mitochondrial genomes of other leuciscid fishes, to clarify the phylogenetic position of *P. grumi* within Leuciscidae. This study provides new molecular evidence for its taxonomic status and lays a foundation for resource conservation, population genetic diversity studies, and analysis of adaptive evolutionary mechanisms in *P. grumi*.

## 2. Materials and Methods

### 2.1. Sample Collection and Genomic DNA Extraction

A single adult female individual of *P. grumi* was collected in July 2024 from Dacao Lake in Turpan, Xinjiang, China. The sample was immediately frozen in liquid nitrogen and stored at −80 °C. Total genomic DNA was extracted from muscle tissue using an animal tissue DNA extraction kit. DNA integrity was assessed by electrophoresis on 1% agarose gels. DNA purity and concentration were evaluated by measuring the OD_260_/OD_280_ ratio using a Nanodrop spectrophotometer (Thermo Fisher Scientific, Wilmington, DE, USA) and a Qubit DNA Assay Kit on a Qubit 3.0 Fluorometer (Invitrogen, Carlsbad, CA, USA), following the manufacturer’s instructions. All experimental procedures involving animals were approved by the Animal Welfare and Ethics Committee of Xinjiang Agricultural University.

### 2.2. High-Throughput Sequencing and Assembly of the Mitochondrial Genome

Qualified DNA samples were sent to Novogene Co., Ltd. (Beijing, China) for high-throughput sequencing. A 350 bp short-insert library was constructed and sequenced on the Illumina NovaSeq X Plus platform (Illumina, San Diego, CA, USA). Raw reads were filtered and trimmed using fastp v 0.23.1 [31] to remove low-quality reads and adapter sequences. The mitochondrial genome was assembled using GetOrganelle v1.7.7.1 [32] with the animal mitochondrial database (option: -F animal_mt). A multi-k-mer strategy (-k 21,35,45,65,85) was applied to improve sequence characteristics across different mitochondrial regions and improve assembly contiguity. The length of the assembled sequence was calculated using SeqKit v2.3.1 [33]. Assembly graphs were visualized with Bandage v0.9.0 [34], confirming a single circular structure without gaps or branches. Finally, BLAST v2.17.0 [35] alignment verified a 100% identical overlapping region between the start and end of the assembly, further validating its circular conformation. The complete mitochondrial genome was annotated and visualized using Chloroplot [36] via the MitoFish online platform v2026.04 (http://mitofish.aori.u-tokyo.ac.jp/, accessed on 28 January 2026).

### 2.3. Mitochondrial Genome Annotation and Characteristic Analysis

The mitochondrial genome was initially annotated using the MITOS Web Server (http://mitos2.bioinf.uni-leipzig.de/index.py, accessed on 10 February 2026) [37] and MitoFish v2026.04 [38]. The secondary structures of tRNA genes were predicted using tRNAscan-SE v2.0 (http://lowelab.ucsc.edu/tRNAscan-SE/, accessed on 27 February 2026) [39], and the predicted open reading frames (ORFs) were verified using ORFfinder (https://www.ncbi.nlm.nih.gov/orffinder/, accessed on 2 March 2026). Manual correction and final annotation of all 37 genes were performed in Geneious Prime v2026.0.2 [40], with reference to the mitochondrial genome of the closely related species *P. phoxinus* (GenBank: NC_020358.1). Base composition and content, skewness, and relative synonymous codon usage (RSCU) of the mitochondrial genome were analyzed using PhyloSuite v.1.2.3 [41]. The YN model [42] of KaKs_Calculator 2.0 [43] was used to calculate the nonsynonymous substitution rate (*Ka*), synonymous substitution rate (*Ks*), and *Ka/Ks* ratio of the 13 PCGs of *P. phoxinus*, with four other leuciscid species (*P. bigerri*, *P. csikii*, *P. phoxinus*, and *P. ujmonensis*) included for comparative selection pressure analysis. The final mitochondrial genome sequence of *P. grumi* has been deposited in the GenBank database under the accession number PZ250837.1.

### 2.4. Molecular Phylogenetic Tree Construction

A total of 67 Leuciscidae species, covering all major subfamilies, were selected as the ingroup based on previously published phylogenetic studies of leuciscid fishes [44]. To ensure robust rooting, two distantly related cypriniform species, *Cobitis striata* (Cobitidae) and *Cyprinus carpio* (Cyprinidae), were designated as the outgroup. Phylogenetic analyses were performed using two independent datasets for complementary analytical purposes: the concatenated nucleotide sequences of the 13 protein-coding genes (PCGs) and, separately, the single mitochondrial *Cytb* gene. We selected the single *Cytb* gene for phylogenetic reconstruction mainly for the following reasons: (1) *Cytb* is the most classic and widely accepted molecular marker for species delimitation and phylogenetic research of leuciscid fishes; (2) an independent *Cytb*-based tree enables direct horizontal comparison with previous studies on Leuciscidae phylogeny; (3) combined results from two datasets can mutually verify the phylogenetic position of *P. grumi* and improve the reliability of phylogenetic inference. The complete mitochondrial genome sequences of all selected species were obtained from NCBI database (Table S1).

Both maximum likelihood (ML) and Bayesian inference (BI) methods were employed to ensure the reliability of the phylogenetic relationships. The ML tree was constructed using IQ-TREE v3.0.1 [45]. Multiple sequence alignment was performed using the MAFFT v7.525 [46] algorithm, and the optimal nucleotide substitution model was automatically selected by ModelFinder [47] during tree construction. Branch support was assessed with 1000 ultrafast bootstrap replicates and 1000 SH-aLRT tests, with other parameters set to default values. The BI tree was constructed using MrBayes v3.2.7 [48]. Based on ModelFinder results, the GTR+G model was selected as the optimal nucleotide substitution model. Markov chain Monte Carlo (MCMC) parameters were set as follows: two independent runs, each with four Markov chains for 15,000,000 generations, sampling every 1000 generations, and outputting diagnostic information every 1000 generations. The MCMC analysis was considered to have reached convergence when the average standard deviation of split frequencies was below 0.01. After the iteration was completed, the first 25% of the trees were discarded as burn-in samples, and the remaining 75% of effective sampled trees were used to construct the allcompat consensus tree. The support strength of each node was evaluated by posterior probability (PP) [49]. The same ML and BI procedures described above were applied to the *Cytb* dataset to compare the phylogenetic signal of the single gene with that of the concatenated 13 PCGs. All phylogenetic trees were visualized and annotated using iTOL v7 (https://itol.embl.de/, accessed on 1 March 2026) [50], with bootstrap values for ML trees and posterior probabilities for BI trees indicated on the branches.

### 2.5. Calculation of Genetic Distances

In this study, the concatenated sequences of 13 PCGs from 31 species were aligned using MAFFT v7.525 [46]. Insertion–deletion sites and ambiguous bases were trimmed to ensure analytical quality. Pairwise genetic distances between species were calculated based on the Kimura 2-parameter (K2P) model [51], and a lower triangular genetic distance matrix was constructed. This approach allowed for quantitative evaluation of the levels of genetic differentiation among *P. grumi* and its closely related groups.

## 3. Results

### 3.1. Structural Characteristics of the Mitochondrial Genome

**Figure 1:**
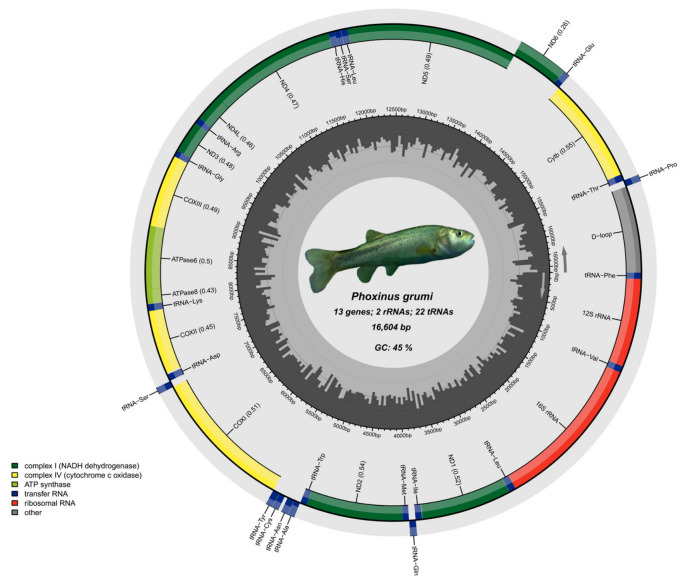
Mitochondrial genome map of *P. grumi*. Genes on the heavy (outer) and light (inner) strands are shown with arrows indicating transcription direction. The innermost circle displays the GC content across the entire mitochondrial genome. Regions with higher GC percentages are represented by deeper peaks.

The complete mitochondrial genome of *P. grumi* is a double-stranded circular DNA molecule with a total length of 16,604 bp. It contains the typical 37 genes (13 PCGs, two rRNA genes, 22 tRNA genes) and a 933 bp non-coding region known as the control region. The light strand encodes nine of these genes, including one PCG and eight tRNA genes; the heavy strand encodes 28 genes, consisting of 12 PCGs, 14 tRNA genes, and two rRNA genes (Figure 1, Table 1). Both gene overlaps and intergenic spacers were observed in the mitochondrial genome of *P. grumi*: there are five gene overlap regions, ranging in size from 1 to 7 bp, with a total length of 21 bp and with the longest overlaps located between *ATP8* and *ATP6* and between *NAD4L* and *NAD4*; there are 13 intergenic spacer regions, ranging in size from 1 to 32 bp, with a total length of 62 bp and with the longest spacer located between *tRNA-Asn* and *tRNA-Cys*.

### 3.2. Base Composition and Skewness of the Mitochondrial Genome

The A, T, C, and G base contents in the complete mitochondrial genome of *P. grumi* are 27.7%, 27.2%, 26.7%, and 18.4%, respectively (Table 2). The AT base content is 54.9%, while the GC base content is 45.1%, showing a clear AT bias, consistent with the base bias in typical freshwater fish. Different codons exhibit a clear base-composition preference. At the first codon position, the base content in descending order is G, A, C, T; at the second position, T, C, A, G; and at the third position, A, C, T, G. The AT content at the second and third codon positions (58.5%) is the highest, while that at the first codon position (47.5%) is the lowest. The total length of the 13 protein-coding genes in the mitochondrial genome of *P. grumi* is 11,412 bp, with AT content ranging from 51.2% (*ND1*) to 59.3% (*ATP8*), and all genes exhibit higher AT than GC content. However, different genes show distinct base preferences: the AT skew is positive only for atp8, indicating a preference for A, whereas all other genes show negative AT skew, indicating a preference for T. Furthermore, the AT-skew value for the whole mitochondrial genome is positive (0.008), whereas the GC-skew value is negative (−0.184), indicating that the A base content is higher than the T base content, while the C base content is higher than the G base content (Table 2).

### 3.3. Protein-Coding Genes and Relative Synonymous Codon Usage of the Mitochondrial Genome

The total length of the 13 PCGs in the mitochondrial genome of *P. grumi* is 11,412 bp, accounting for 68.73% of the total mitochondrial genome length. Among PCGs, *ND5* is the longest (1836 bp), while *ATP8* is the shortest (165 bp). The PCGs in the mitochondrial genome have an uneven base distribution: the AT content accounts for 54.8% of the total protein-coding gene sequences, while GC accounts for 45.2%, with AT significantly exceeding GC. Among all PCGs, *ATP8* has the highest A base content at 34.5%, while *ND6* has the lowest at 15.3%. Similarly, *ND6*, located on the light strand, has the highest T base content at 36.2%, while *ATP8* has the lowest at 24.8%. *ND6* also has the highest G base content at 33.5%, while *ATP8* has the lowest at 12.7%. *ND2* has the highest C base content at 31.6%, while *ND6* has the lowest at 14.9%. In addition, the 13 PCGs have an overall AT skew and GC skew of −0.077 and −0.201, respectively (Table 2). All PCGs show negative GC skew, except for the light-strand gene *ND6*, which exhibits a positive GC skew. Similarly, all PCGs display negative AT skew except *ATP8*, which shows positive AT skew.

**Figure 2:**
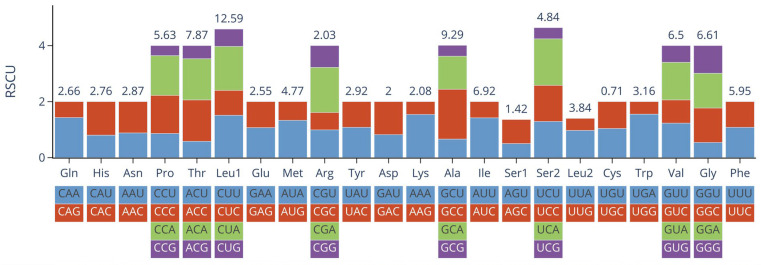
Relative synonymous codon usage (RSCU) of PCGs in the mitogenome of *P. grumi*. Codons are grouped by amino acid on the *x*-axis, with corresponding RSCU values shown on the *y*-axis. Different colors are used to distinguish synonymous codons within each amino acid and do not represent specific biological categories.

The 13 PCGs of the *P. grumi* encode a total of 3798 amino acids, with leucine (Leu) being the most abundant (12.59%). Of the 13 protein-encoding genes, 12 use the typical ATG start codon; the *COX1* gene uses the GTG start codon. The *ND1*, *COX1*, *ATP8*, *ND4L*, *ND5*, and *ND6* genes use TAA as the stop codon, while *ND2*, *COX2*, *ATP6*, *COX3*, *ND3*, *ND4*, and *Cytb* use an incomplete TA/T stop codon, consistent with the stop codons commonly used in fish mitochondrial genomes. Analysis of relative synonymous codon usage (RSCU) revealed 29 preferred codons. The most frequently used codons include AUU (Ile), CUA (Leu1), and GCC (Ala). GCC has the highest RSCU value (1.78), and CCG the lowest (0.36) (Figure 2).

### 3.4. Selective Pressure Analysis of the Mitochondrial Genome

**Figure 3:**
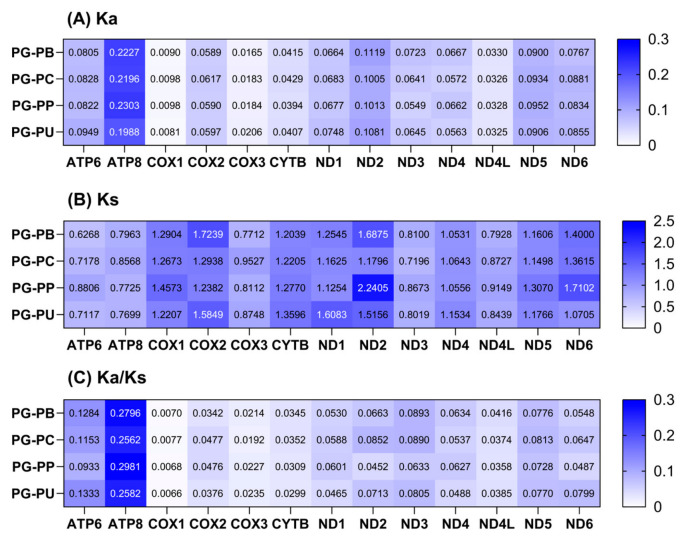
*Ka*, *Ks*, and *Ka/Ks* values of PCGs in the mitochondrial genomes of five Leuciscidae species. The abbreviations used are as follows: PG represents *P. grumi*, PB represents *P. bigerri*, PC represents *P. csikii*, PP represents *P. phoxinus*, and PU represents *P. ujmonensis*.

The nonsynonymous (*Ka*) and synonymous (*Ks*) substitution values and their ratio were calculated for each PCG in selected species. The results show *Ka/Ks* values less than 1 for all mitochondrial PCGs in five species, indicating that the mitochondrial genomes of *P. grumi* and four closely related species underwent purifying selection. *ATP8* shows the highest divergence across all species (*Ka/Ks* = 0.2562–0.2981) and represents the fastest-evolving gene, whereas *COX1* exhibits the lowest divergence (*Ka/Ks* = 0.0066–0.0077) and is considered an extremely conserved core gene (Figure 3). The lowest average *Ka/Ks* (0.0683) is between *P. grumi* and *P. phoxinus*, indicating that their phylogenetic relationship is the closest; the average *Ka/Ks* (0.0732) between *P. grumi* and *P. bigerri* is slightly larger. In terms of grouping, 77% (10/13) of the mitochondrial genes of *P. grumi* exhibit moderate evolutionary rates (0.02 ≤ *Ka/Ks* < 0.1); only 15% (2/13) are classified as fast-evolving genes (*ATP8*, *ATP6*), and only one (8%; 1/13) is considered an extremely conserved gene (*COX1*) (Figure 3). This comparative analysis highlights the diverse evolutionary dynamics within the mitochondrial genome of *Phoxinus*.

### 3.5. RNA Genes and Non-Coding Regions of the Mitochondrial Genome

**Figure 4:**
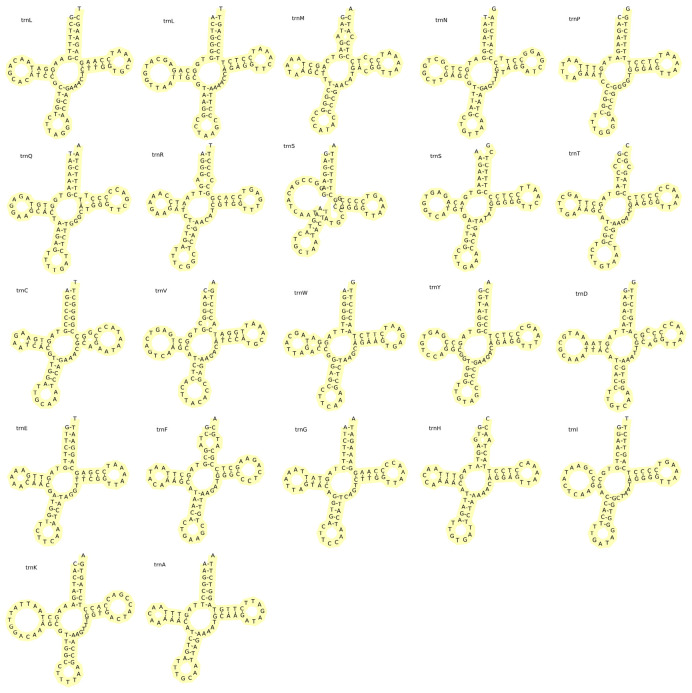
Secondary structures of tRNA genes in the mitogenome of *P. grumi*.

The 22 tRNA genes in the complete mitochondrial genome of *P. grumi* range in length from 68 to 72 bp, with the longest sequence being *tRNA-Lys* and the shortest tRNA-Cys, for a total length of 1563 bp. Eight of these tRNA genes—*tRNA-Gln*, *tRNA-Ala*, *tRNA-Asn*, *tRNA-Cys*, *tRNA-Tyr*, *tRNA-Ser1*, *tRNA-Glu*, and *tRNA-Pro*—are encoded by the L-strand, while the other 14 tRNA genes are encoded by the H-strand. The AT content of these tRNA genes is 54.5%, significantly higher than the GC content, and both AT skew (0.046) and GC skew (0.042) are positive (Table 2). Furthermore, *tRNA-Leu* and *tRNA-Ser* genes exhibit double-copy characteristics, while the remaining tRNA genes are single-copy. Only the *tRNA-Ser1* gene lacks the dihydrouracil (DHU) arm, which is replaced by a simple loop; the remaining 21 tRNA genes all form a complete cloverleaf structure. We also found multiple non-Watson–Crick base mismatches in the stem region, primarily G–A, A–C, and U–U pairings. Such mismatches—common features of mitochondrial tRNAs in fishes and other vertebrates—may be repaired through post-transcriptional modification or RNA editing to ensure the normal function of the tRNAs (Figure 4).

The two rRNA genes in the complete mitochondrial genome of *P. grumi* have a total length of 2645 bp and are both located on the H-strand, with no gene gaps or overlaps. The *12S rRNA* gene is located between *tRNA-Phe* and *tRNA-Val* and is 955 bp long, while *16S rRNA* is located between *tRNA-Val* and *tRNA-Leu* and is 1690 bp long. The AT content is higher than the GC content in both *12S rRNA* (50.1% AT and 49.8% GC) and 16S rRNA (54.4% AT and 45.7% GC). The AT skew and GC skew of the combined rRNA genes are 0.224 and −0.023, respectively. The AT-skew value of 16S rRNA (0.224) is higher than that of *12S rRNA* (0.19), and both are positive, indicating a relatively high abundance of adenine (A).

Similarly to other fish mitochondrial genomes, the mitochondrial genome of *P. grumi* contains two non-coding regions. One region is called the L-chain replication initiation region, also known as the OL region, which is mainly responsible for initiating L-chain replication. The other region is called the control region, also known as the D-loop region, which is 933 bp long and located between the *tRNA-Pro* and *tRNA-Phe* genes.

### 3.6. Phylogenetic Analysis

**Figure 5:**
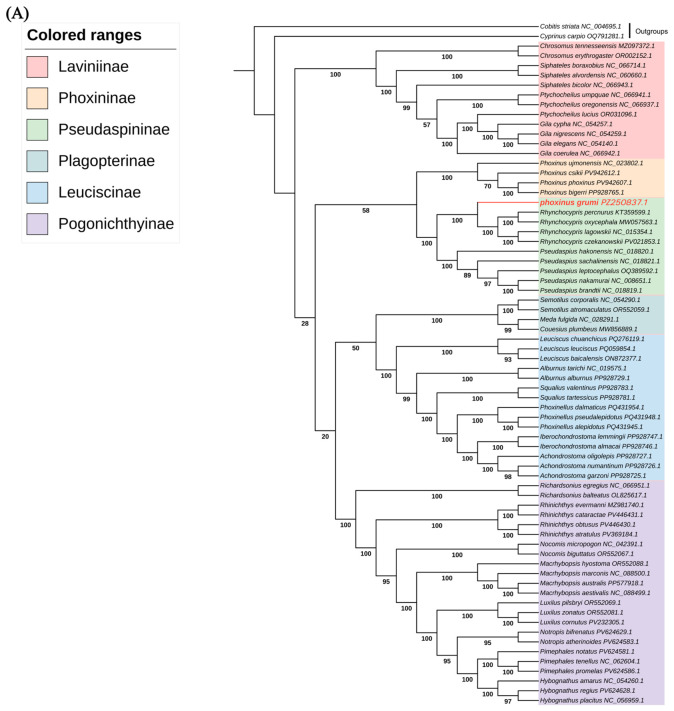

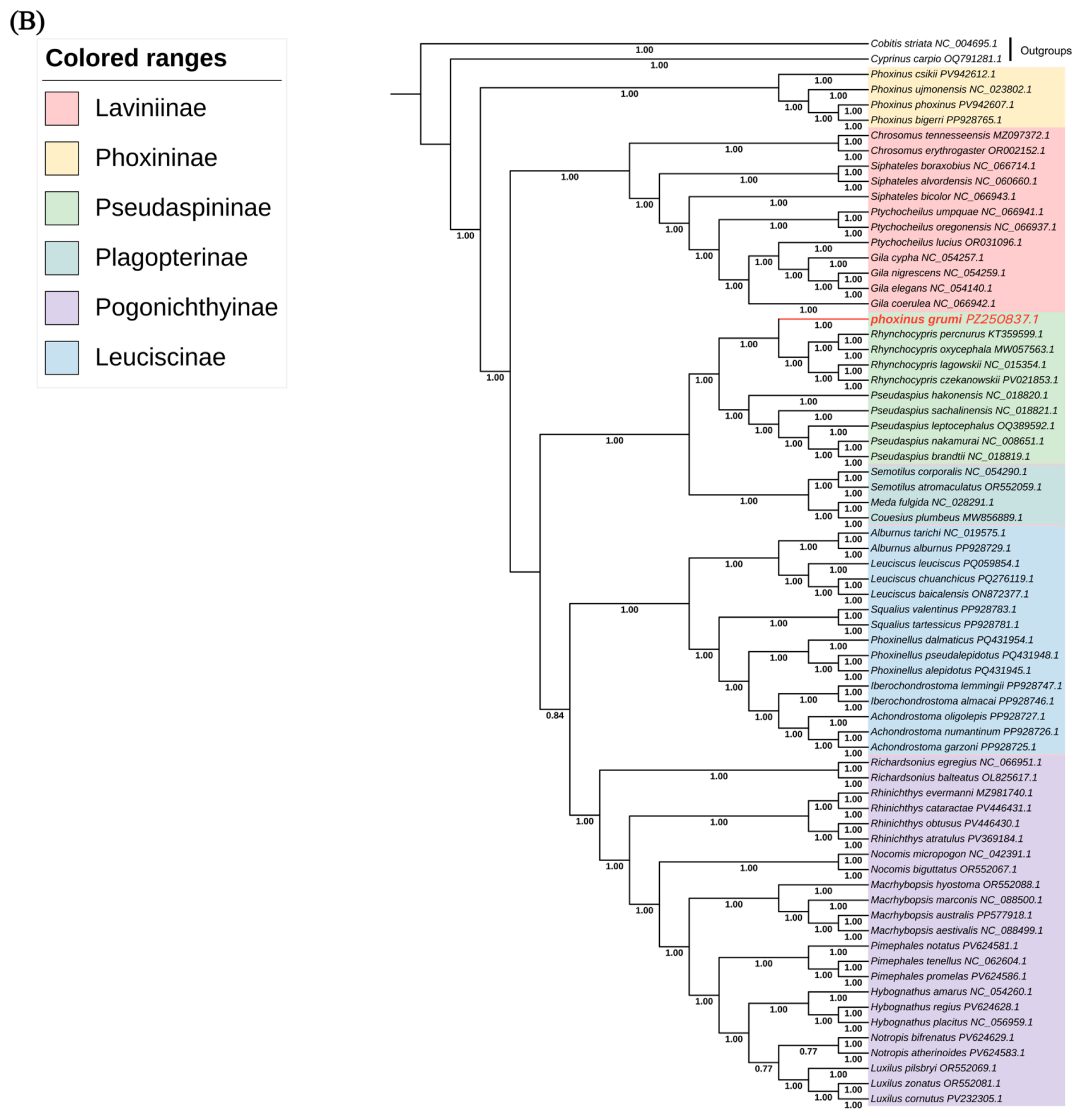
Phylogenetic trees of Leuciscidae constructed based on the nucleotide sequences of 13 PCGs. (**A**) ML tree constructed using IQ-TREE. (**B**) BI tree constructed using MrBayes. The numbers below the branches represent confidence levels. Different subfamilies are indicated by colored ranges listed to the left of the trees. The position of *P. grumi* is highlighted in red for clarity.

To clarify the phylogenetic position and evolutionary relationships of *P. grumi* within Leuciscidae, we reconstructed phylogenetic trees based on two datasets: the concatenated sequences of the 13 PCGs and the mitochondrial *Cytb* gene sequence. Phylogenetic trees were constructed using two methods, ML (Figure 5A) and BI (Figure 5B), covering the subfamilies Laviniinae, Leuciscinae, Phoxininae, Plagopterinae, Pogonichthyinae, and Pseudaspininae. The phylogenetic trees constructed by the two methods are largely consistent, supporting the accuracy and reliability of the results. With *C. striata* and *C. carpio* designated as outgroups, 68 species from the subfamilies Laviniinae, Leuciscinae, Phoxininae, Plagopterinae, Pogonichthyinae, and Pseudaspininae cluster into six clades, forming sister groups, with each clade node achieving high support values. Both ML and BI trees consistently recover *P. grumi* as a member of the subfamily Pseudaspininae, where it forms a well-supported clade with species of the genus *Rhynchocypris*. This molecular evidence suggests that *P. grumi* is more closely related to these lineages than to other *Phoxinus* genus members, which are grouped within the subfamily Phoxininae. Notably, in the ML tree based on the 13 PCGs, the bootstrap support for the divergence between Phoxininae and Pseudaspininae was relatively low, indicating weak support for a sister relationship between these two subfamilies. However, the BI tree suggested that Pseudaspininae is more closely related to Plagopterinae than to Phoxininae.

Additionally, phylogenetic trees based on the mitochondrial *Cytb* gene were constructed using the same ML (Figure S1) and BI (Figure S2) methods. The topology of the *Cytb* trees is generally congruent with that of the 13-PCG trees. Phylogenetic analysis based on the mitochondrial PCGs revealed that *P. grumi* belongs to the Pseudaspininae subfamily, rather than the typical Phoxininae clade. In both the ML and BI trees, *P. grumi* forms a strongly supported sister relationship with species of the genus *Rhynchocypris*. A similar topology is recovered in the *Cytb*-based trees, in which *P. grumi* also clusters within the Pseudaspininae clade with high support. This relationship was strongly supported in both ML and BI analyses across the two datasets, suggesting a closer evolutionary affinity between *P. grumi* and *Rhynchocypris* species and thus confirming the phylogenetic position of *P. grumi* within the subfamily Pseudaspininae.

### 3.7. Genetic Distance Analysis

To further quantify the phylogenetic divergence between *P. grumi* and its closely related species, we calculated the Kimura two-parameter (K2P) genetic distances based on the concatenated nucleotide sequences of the 13 PCGs from 31 species. The resulting distance matrix revealed a clear divergence pattern that was highly consistent with the phylogenetic tree topology. Among all taxa, the smallest K2P genetic distances, ranging from 0.1620 to 0.1703, were between *P. grumi* and *Rhynchocypris*, indicating that these two species have the closest genetic relationship. The distances between *P. grumi* and *Pseudaspius* congeners were slightly higher, varying from 0.1832 to 0.1857, which represents a typical level of intergeneric differentiation. Notably, the genetic distances between *P. grumi* and *Phoxinus* species reached 0.2475–0.2558, which is much higher than the general differentiation threshold for congeneric species. This evidence suggests that *P. grumi* should not be placed in the narrowly defined genus *Phoxinus* and validates its taxonomic status as an independent evolutionary lineage. The genetic distances between *P. grumi* and two other Leuciscidae subfamilies, Laviniinae and Plagopterinae, exceeded 0.22. Furthermore, the genetic distances to the outgroups *C. carpio* and *C. striata* were 0.2640 and 0.2949, respectively, and increased gradually with the distance of phylogenetic relatedness, which conforms to the evolutionary pattern of vertebrate mitochondrial DNA (Table S2). These results indicate that *P. grumi* exhibits the closest genetic relationship with members of the Pseudaspininae subfamily, particularly the genus *Rhynchocypris*, supporting its classification within this subfamily rather than the traditional genus *Phoxinus* of the Phoxininae subfamily. This conclusion is highly consistent with the topological structures of the ML and BI phylogenetic trees.

## 4. Discussion

### 4.1. Genomic Features of the Mitochondrial Genome of P. grumi

In this study, the complete mitochondrial genome of *P. grumi* was first successfully sequenced and annotated. It is a typical covalently closed circular double-stranded DNA molecule with a total length of 16,604 bp, containing 37 core genes (13 PCGs, 22 tRNA genes, and two rRNA genes) and one non-coding control region. This genome composition pattern is highly conserved in Leuciscidae fishes and is completely identical to that in species such as *P. phoxinus* [52], *Rhinichthys* cf. *lagowskii* [53], and *Leuciscus chuanchicus* [54], as well as other bony fishes such as *Gymnocypris przewalskii* [55] and *Arothron stellatus* [56], reflecting the high conservation of the core mitogenome composition in bony fishes.

No obvious gene rearrangement was observed in the mitochondrial genome of *P. grumi*, and its gene arrangement order is consistent with that in most Leuciscidae taxa. This highly conserved gene arrangement pattern is common in bony fish and is considered a typical feature in the evolution of vertebrate mitochondrial genomes [12]. In addition, there are a few gene overlap regions and spacer regions in the mitochondrial genome of *P. grumi*. The overlaps mainly occur in the *ATP8-ATP6* and *ND4L-ND4* (7 bp) regions. These overlapping phenomena are common in the sequences of other Leuciscidae species [57]. The total length of the spacer regions is relatively short, reflecting the evolutionary feature of a compact mitochondrial genome structure. Therefore, the mitochondrial genome structure of *P. grumi* is consistent with that of other Leuciscidae fishes as a whole, further indicating that this group has high structural stability at the mitochondrial genome level.

### 4.2. Nucleotide Composition and Skew Characteristics of the Mitochondrial Genome of P. grumi

The mitochondrial genome of *P. grumi* exhibits nucleotide composition characteristics typical of Leuciscidae fishes—namely, a marked AT bias and strand asymmetry. In the heavy strand of the complete mitochondrial genome of *P. grumi*, the AT content is significantly higher than the GC content. This bias is widespread across Leuciscidae and even Cypriniformes fishes and mainly arose from the combined effects of mutational pressure and selective pressure [57]. Specifically, the overall nucleotide composition is A 27.7%, T 27.2%, C 26.7%, and G 18.4%, with a total AT content of 54.9%. The mitochondrial genome of *P. grumi* shows a positive AT skew and a negative GC skew, which is consistent with observations in most teleost fishes and conforms to the typical asymmetry of the heavy strand in vertebrate mitochondrial genomes. This skew pattern is generally attributed to mutational pressure caused by prolonged single-stranded exposure of the light strand during mtDNA replication, in which cytosine (C) is prone to deamination to thymine (T), resulting in relatively low G content and relatively elevated A content on the heavy strand.

Nucleotide composition significantly differs among genomic regions. Of the PCGs, rRNA genes, and tRNA genes, PCGs have the highest AT content, followed by tRNA genes, while rRNA genes have the lowest. Nucleotide composition also differs among PCGs. Genes such as *ATP8* and *COX2* have relatively high AT content, whereas conserved genes such as *ND1* and *ND2* have relatively low AT content, which is associated with the evolutionary rate and functional conservation of the genes [9,58]. Comparative analysis with other Leuciscidae fishes further confirmed the conservation of the nucleotide composition and skew characteristics of *P. grumi*. For example, the nucleotide composition of the heavy strand of *P. phoxinus ujmonensis* is A 28.50%, T 28.24%, C 25.32%, and G 17.94%, which also shows obvious AT bias and strand asymmetry [59]; among species of the genus Leuciscus, the overall genome nucleotide composition of *Leuciscus leuciscus* is A 28.7%, T 26.4%, C 27.0%, and G 17.9%, and its AT-skew and GC-skew patterns are highly consistent with those of *P. grumi* [57]. These comparative results indicate that although *P. grumi* has long been adapted to the unique stream habitats of the arid Turpan Basin, the nucleotide composition and skew characteristics of its mitochondrial genome do not deviate significantly from the ancestral state of Leuciscidae. This suggests the strong evolutionary conservation of these characteristics, potentially because of the stable demand for efficient replication and transcription of mitochondrial DNA. In addition, nucleotide composition and skew characteristics do not significantly differ between *P. grumi* and species of *Rhynchocypris*, which is consistent with the close genetic relationship between them revealed by phylogenetic analysis [44,54].

### 4.3. Characteristics of Protein-Coding Genes in the Mitochondrial Genome of P. grumi

Mitochondrial PCGs play vital roles in maintaining mitochondrial oxidative phosphorylation and energy metabolism. They also serve as important molecular markers for investigating animal phylogeny and molecular evolution. The mitochondrial genome of *P. grumi* contains the typical 13 protein-coding genes with a total length of 11,412 bp, accounting for 68.73% of the entire mitogenome. This proportion is highly consistent with that in other fishes in the family Leuciscidae. The arrangement of PCGs in *P. grumi* conforms to the typical structure of vertebrate mitochondrial genomes: 12 PCGs are encoded on the H-strand, while only the *ND6* gene is located on the L-strand. This pattern is observed in most bony fishes. The lengths of the 13 PCGs range from 165 bp (*ATP8*) to 1836 bp (*ND5*) and are largely consistent with those in *P. phoxinus* [52], *R.* cf. *lagowskii* [53], *L. chuanchicus*, and other related species. There are only 1–3 bp differences at the ends of a few genes, which are mainly caused by the use of incomplete stop codons.

The start codons of the 13 PCGs in *P. grumi* are significantly conserved. Twelve genes use the standard ATG as the start codon; only *COX1* employs GTG as the start codon. This feature is common in species of the genera *Phoxinus* and *Leuciscus* in the family Leuciscidae [54,57]. In other groups of Cypriniformes, species such as *Pseudorasbora parva* [60] and *G*. *przewalskii* [61] also use GTG as the start codon for *COX1*, indicating that GTG is widely used as an alternative start codon in Cypriniformes fishes, and its use is gene-specific, which is closely related to the transcriptional regulatory mechanism of mitochondrial DNA [8,55]. Mitochondrial PCGs in *P. grumi* exhibit diversity in their stop codons, including complete stop codons (TAA) and incomplete stop codons (TA, T). Incomplete stop codons can form complete stop signals through post-transcriptional polyadenylation [61,62], which is a common mechanism in fish mitochondrial genomes. Specifically, *ND1*, *COX1*, *ATP8*, ND4L, ND5, and ND6 use TAA as the stop codon, whereas *ND2*, *COX2*, *ATP6*, *COX3*, *ND3*, *ND4*, and *Cytb* use TA or T as incomplete stop codons. This pattern is also prevalent in other Leuciscidae species [59,63] and is presumed to be associated with transcriptional efficiency and functional adaptation of the genes [8,64].

Relative synonymous codon usage (RSCU) analysis is an important approach for investigating codon usage bias in genomes. The results of this study show that the mitochondrial PCGs of *P. grumi* exhibit significant codon usage bias. The RSCU values of codons ending in A/T are all greater than 1, while those of codons ending in G/C are mostly less than 1. This phenomenon is closely related to the high AT content in the overall mitochondrial genome, and such bias is highly similar to that observed in Leuciscidae species, including *L. baicalensis* and *Rutilus rutilus* [3]. Among these, AUU (Ile), CUA (Leu1), and GCC (Ala) are used at relatively high frequencies. Similar codon usage biases have been detected in numerous fish species. For instance, studies on the mitochondrial genome of the cypriniform fish *Onychostoma ovale* revealed that synonymous codons tend to end with A or T, a preference thought to be related to mitochondrial DNA replication and mutation bias [60,65]. In terms of amino acid composition, leucine (Leu), alanine (Ala), and threonine (Thr) are the most frequent in the mitochondrial PCGs of *P. grumi*, where they account for more than 40% of the total amino acids. Therefore, the codon usage bias observed in the PCGs of *P. grumi* conforms to the general pattern of fish mitochondrial genomes.

The *Ka/Ks* ratio serves as a key indicator for evaluating evolutionary selection pressure on PCGs. A *Ka/Ks* value less than 1 indicates that the gene is mainly subjected to purifying selection; a *Ka/Ks* value greater than 1 may indicate positive selection pressure [66]. In this study, all 13 mitochondrial PCGs in *P. grumi* have *Ka/Ks* ratios less than 1, indicating that, overall, they have been subjected to strong purifying selection and are functionally highly conserved. This result is consistent with studies on selection pressure of mitochondrial PCGs in Leuciscidae and other Cypriniformes fishes, indicating that *P. grumi* did not detect obvious positive selection signals during its long-term adaptation to the stream habitat in the arid Turpan Basin but instead ensured the normal functioning of mitochondrial energy metabolism by maintaining functional stability. Notably, evolutionary rates vary among PCGs, reflecting differences in functional constraints (Figure 3). *COX1* (*Ka/Ks* = 0.01) and *COX3* (*Ka/Ks* = 0.02) experienced the strongest selection pressure and the most intense purifying selection. In contrast, *ATP8* (*Ka/Ks* = 0.27) and *ATP6* (*Ka/Ks* = 0.12) exhibited higher *Ka/Ks* ratios, faster evolutionary rates, and weaker purifying selection constraints. Furthermore, previous studies also reported that the *COX1* gene generally evolves slowly, while *ND2* and *ATP8* tend to show higher evolutionary rates [67]. Such variation is related to the functional importance of different genes in the respiratory chain and is widespread among teleost fishes.

### 4.4. Characteristics of the RNA Genes and Non-Coding Region of the Mitogenome of P. grumi

The mitochondrial genome of *P. grumi* contains two rRNA genes, 22 tRNA genes, and one major non-coding control region. These RNA genes, together with the non-coding region, constitute the non-protein-coding portion of the mitochondrial genome, and their structural characteristics are highly consistent with those of other fishes in the family Leuciscidae.

The two rRNA genes were identified as *12S rRNA* and *16S rRNA*, located between *tRNA-Phe* and *tRNA-Val* and between *tRNA-Val* and *tRNA-Leu1*, which is consistent with the conserved pattern of mitochondrial genomes in most vertebrates [12]. The length and nucleotide composition of *12S rRNA* and *16S rRNA* in *P. grumi* are similar to those in closely related species such as *P.* cf. *phoxinus* and *L. leuciscus*, exhibiting typical characteristics of fish mitochondrial rRNAs. Generally, *16S rRNA* is longer than *12S rRNA*, and both genes participate in the assembly of mitochondrial ribosomes, which is essential for mitochondrial protein synthesis. The rRNA genes show slow evolutionary rates and contain highly conserved regions, making them particularly suitable for deep phylogenetic analyses. Numerous fish phylogenetic studies have used *12S rRNA* and *16S rRNA* as molecular markers, combined with other mitochondrial genes, to infer phylogenetic relationships [60]. The high conservation of rRNA genes in *P. grumi* further supports their potential as reliable markers for phylogenetic analysis.

The mitochondrial genome of *P. grumi* contains the 22 typical tRNA genes, ranging from 68 to 72 bp in length. Eight of these tRNA genes are encoded on the L-strand, while the remainder are located on the H-strand, with these genes distributed across different regions of the mitochondrial genome. Secondary structure predictions show that, consistent with most vertebrate lineages, the vast majority of tRNAs can fold into the typical cloverleaf secondary structure, including the amino acid acceptor stem, dihydrouracil stem (DHU stem), anticodon stem, variable loop, and TΨC stem. However, tRNA-Ser lacks the DHU arm, which is a common structural simplification in mitochondrial tRNA molecules [67]. This structural simplification does not impair the basic translational function of the tRNAs, as the mitochondrial translation machinery generally tolerates non-canonical tRNA conformations [62]. This feature is widespread among Leuciscidae and even Cypriniformes fishes and has been reported in *P. phoxinus*, *L. chuanchicus*, and *Rhynchocypris czekanowskii*. In addition, several base mismatches exist in the tRNA genes, a common characteristic of fish mitochondrial tRNAs that does not affect their transport function [67].

Non-coding regions mainly include the control region (D-loop) and the origin of light-strand replication (OL region). The control region is one of the most important non-coding segments in the mitochondrial genome and is the region with the greatest length variation. It typically contains multiple conserved sequence elements involved in the regulation of mitochondrial DNA replication and transcription. In *P. grumi*, the control region is 933 bp long and located between *tRNA-Pro* and *tRNA-Phe*. The length, nucleotide composition, and structural elements of the control region—serving as a key regulatory region for mtDNA replication and transcription—are highly similar to those in other Leuciscidae fishes [9,21]. In comparative mitogenomic analyses of many fish, the control region has been shown to contain similar conserved sequences and has been widely used to investigate lineage divergence and population history [68]. The OL region can form a stable stem-loop structure and acts as the initiation site for light-strand replication [5,9]. Its sequence and structure are also relatively conserved among Leuciscidae fishes.

### 4.5. Phylogenetic Position and Genetic Divergence of P. grumi

Morphological considerations further highlight the significance of this molecular framework. The traditional taxonomy of *P. grumi* has relied heavily on characters such as scale counts, fin-ray numbers, and body proportions. However, these traits are prone to homoplasy and the retention of plesiomorphic features, which can lead to misleading classifications [6,24]. The robust molecular evidence presented here indicates that several morphological traits previously used to assign *P. grumi* to *Phoxinus* are likely not synapomorphies for that genus. The phylogenetic relationships of *P. grumi* within the family Leuciscidae were reconstructed using both ML and BI methods based on the concatenated nucleotide sequences of 13 mitochondrial PCGs, as well as the mitochondrial *Cytb* gene separately. Both the ML and BI trees derived from the two datasets recovered six major monophyletic subfamilies with generally high nodal support: Laviniinae, Phoxininae, Pseudaspininae, Plagopterinae, Pogonichthyinae, and Leuciscinae [6]. The topologies derived from the 13 PCGs and *Cytb* datasets were largely congruent. *C. carpio* and *C. striata* served as outgroups and were stably positioned at the basal node of the tree, further supporting the overall reliability of the phylogenetic framework.

Analyses based on both the concatenated 13 PCGs and the *Cytb* gene consistently placed *P. grumi* within the subfamily Pseudaspininae, rather than the traditionally recognized Phoxininae. In all phylogenetic trees, *P. grumi* formed a monophyletic clade with species of the genus *Rhynchocypris*, along with *Pseudaspius* species, forming the core lineage of Pseudaspininae. This clade was clearly separated from typical *Phoxinus* species, which clustered within the Phoxininae clade. A notable topological discordance was observed in the position of the subfamily Phoxiniinae. In the ML tree based on the concatenated 13 PCGs (Figure 5A), Phoxiniinae appeared as sister to Pseudaspininae, whereas in the BI tree based on the 13 PCGs (Figure 5B) and both ML and BI trees based on the *Cytb* gene (Figures S1 and S2), Pseudaspininae clustered as sister to Plagopterinae. Such incongruence has been frequently reported in previous mitogenome-based studies of Leuciscidae [6]. This result further highlights a well-recognized limitation of mitochondrial genome-based phylogenetic analyses, namely, that mitochondrial markers alone contain insufficient phylogenetic signal to robustly resolve deeper evolutionary relationships [69]. This discordance is likely due to rapid early diversification within these lineages or the limited phylogenetic signal in mitochondrial data to resolve ancient divergences within the family [70]. Previous studies have documented inconsistencies between mitochondrial and nuclear gene trees when analyzing deep phylogenetic relationships across diverse taxonomic groups [71]. These discrepancies may arise from several factors, including incomplete lineage sorting, ancient hybridization, introgression, and differential rates of molecular evolution among genomic compartments [72]. The conflicting topologies observed between mitochondrial and nuclear data in our study, as well as in other fish phylogenetic investigations, are consistent with these documented patterns. To address these limitations, future studies should incorporate nuclear gene datasets alongside mitochondrial genomes to provide more robust phylogenetic estimates. Importantly, despite the instability of deeper subfamily relationships, the phylogenetic position of *P. grumi* within Pseudaspininae and its close relationship with *Rhynchocypris* remain highly stable and strongly supported across all four analyses. These results align with and extend previous phylogenetic studies on Far Eastern phoxinins. Early molecular studies using allozymes and partial mtDNA sequences suggested that *Rhynchocypris* forms a monophyletic group with *Pseudaspius*, distinct from the Eurasian *Phoxinus* clade [24]. Subsequent mitogenome-based analyses further supported the recognition of Pseudaspininae as an independent East Asian lineage while demonstrating that the traditional Phoxininae is non-monophyletic [6]. Our study, utilizing both the concatenated 13 PCGs and the *Cytb* gene, provides additional robust evidence confirming that *P. grumi* belongs to this East Asian Pseudaspininae lineage rather than the Eurasian *Phoxinus* clade.

The phylogenetic results were validated by calculating Kimura two-parameter (K2P) genetic distances based on the concatenated 13 PCGs from 31 Leuciscidae species (Table S2). The analysis revealed a clear divergence pattern that is highly consistent with the tree topologies from both datasets. The genetic distances from *P. grumi* to *Rhynchocypris* species were the smallest (0.1620–0.1703), followed by distances to *Pseudaspius* species (0.1832–0.1857). In contrast, distances to core *Phoxinus* species were substantially higher (0.2475–0.2558), exceeding typical intergeneric differentiation levels within Leuciscidae. Distances to other subfamilies generally exceeded 0.22, while distances to the outgroups ranged from 0.2640 to 0.2949. These K2P distance patterns strongly support the phylogenetic placement of *P. grumi* closer to the Rhynchocypris lineage rather than the core Phoxinus clade, further reinforcing the taxonomic distinction between Rhynchocypris and Phoxinus. Sakai et al. (2006) [24], using allozyme and partial mitochondrial 16S rRNA analyses, demonstrated that *Rhynchocypris* species form a monophyletic group distinct from *P. phoxinus*, instead clustering with dace-like genera such as *Tribolodon* and *Pseudaspius*. They concluded that the traditional broad concept of *Phoxinus* is paraphyletic [24]. More recent studies using mitochondrial protein-coding genes have yielded congruent results. Kusznierz et al. (2023) reported a *Cytb* genetic distance of approximately 0.24 between Phoxinus and *Rhynchocypris*, interpreted as indicative of generic-level divergence (consistent with mean intergeneric *Cytb* distances of ~0.19 in Cypriniformes) [73]. This value is remarkably similar to the 0.2475–0.2558 observed in the present study between *P. grumi* and core *Phoxinus* species based on the concatenated 13 PCGs. At the subfamily level, our findings align with the comprehensive Leuciscidae phylogeny proposed by Schönhuth et al. (2018) [6]. Using multiple mitochondrial and nuclear markers, they recovered a distinct Far East Asian (FEA) clade (Pseudaspininae) that includes several genera traditionally associated with phoxinins, while the core Eurasian *Phoxinus* formed a separate Phoxininae clade. Neither the traditional phoxinin group nor a broad *Phoxinus s.l.* was monophyletic [6]. The relatively small distances between *P. grumi* and *Rhynchocypris/Pseudaspius* (0.1620–0.1857) in our dataset are consistent with an affinity to this Far East Asian lineage. In Cypriniformes, K2P distances of 0.15–0.25 or higher for mitochondrial genomes (especially concatenated PCGs) are frequently regarded as indicative of intergeneric differentiation. The substantially elevated distances to core *Phoxinus* (>0.247) in the present study therefore exceed many reported intergeneric thresholds, further supporting the distinction between *Rhynchocypris* and *Phoxinus* as separate genera, a view increasingly endorsed by earlier allozyme/mtDNA work and recent multi-gene analyses.

As a cold-water fish endemic to the extremely arid rivers of the Turpan Basin in northwest China, *P. grumi* provides valuable insights into the biogeographic history and adaptive radiation of Leuciscidae in East and Central Asia. Previous large-scale mitogenomic studies have suggested that ancestral leuciscids originated in Europe during the Late Cretaceous, dispersed to North America, and subsequently colonized East Asia via the Beringia land bridge, with subsequent regional diversification driven by tectonic movements and climatic fluctuations [6,11]. Pseudaspininae, containing the genera *Rhynchocypris*, *Pseudaspius*, and *Oreoleuciscus*, is an endemic monophyletic subfamily in East Asia [24,74]. The moderate genetic divergence observed between *P. grumi* and *Rhynchocypris* species, combined with the former’s isolated distribution in the extreme arid habitats of the Turpan Basin, suggests that long-term geographical isolation has played a key role in shaping its distinct evolutionary trajectory. This prolonged isolation, together with strong selective pressures from high temperatures, low water availability, and desert river conditions, likely promoted local adaptations to this harsh environment. Consequently, this study confirms that *P. grumi* does not belong to Phoxininae or the genus *Phoxinus* but instead represents a member of the East Asian endemic subfamily Pseudaspininae, with the closest phylogenetic affinity to the genus *Rhynchocypris*. To better resolve the systematics of Pseudaspininae and identify true synapomorphies, future integrative taxonomic studies should combine detailed morphological re-examination of type specimens with both mitogenomic and nuclear genomic data.

## 5. Conclusions

We determined the complete mitochondrial genome sequence of *P. grumi* for the first time, systematically elucidating its structural features and clarifying its phylogenetic position within the family Leuciscidae. The mitochondrial genome of *P. grumi*, with a total length of 16,604 bp, contains 13 PCGs, 22 tRNA genes, two rRNA genes, and one control region. The gene order conforms to the typical vertebrate pattern, with no gene rearrangements observed. Most tRNAs fold into the classic cloverleaf secondary structure, the exception being *tRNA-Ser1*, which lacks the DHU arm. The base composition exhibits a significant AT bias, with chain asymmetry between the positive AT skew and negative GC skew. The start and stop codon usage patterns of the 13 PCGs are conserved, and RSCU shows a preference for A/T-ending codons. The *Ka/Ks* values are all significantly less than 1, indicating that all PCGs are subject to strong purifying selection pressure. These structural features are highly consistent with those of other species in the family Leuciscidae, demonstrating the conservation of the mitochondrial genome in fish evolution and providing a molecular basis for further research on the energy metabolism mechanism of *P. grumi* as it adapted to the arid stream habitat of the Turpan Basin.

Phylogenetic analyses based on the concatenated sequences of the 13 mitochondrial PCGs and the *Cytb* gene were conducted using both ML and BI methods, which yielded largely congruent topologies. Both datasets consistently supported the placement of *P. grumi* within the subfamily Pseudaspininae, where it formed a strongly supported monophyletic clade with species of the genus *Rhynchocypris*, rather than clustering with core *Phoxinus* species of the subfamily Phoxininae as traditionally classified. This inference was further supported by Kimura two-parameter (K2P) genetic distance analyses, which revealed the smallest distances between *P. grumi* and *Rhynchocypris* species (0.1620–0.1703), markedly smaller than those observed between *P. grumi* and *Phoxinus* species (0.2475–0.2558). Together, these results strongly support the placement of *P. grumi* within the East Asian Pseudaspininae lineage and help clarify its taxonomic position, which has long been debated. By integrating multiple mitochondrial datasets, this study provides additional mitogenomic evidence for understanding phylogenetic relationships within Leuciscidae and contributes to a more informed interpretation of this family’s evolutionary history and diversification in East and Central Asia.

In summary, this study not only expands the mitochondrial genomic resources for endemic fishes in northwestern China but also provides a molecular framework for re-evaluating the taxonomic status of *P. grumi*. These findings are relevant for future conservation genetics and for understanding how freshwater fishes adapt to extreme arid environments. However, given the limitations of mitochondrial data, future studies incorporating nuclear genomic markers and population-level sampling will be essential to further resolve evolutionary relationships and elucidate the mechanisms underlying local adaptation in this species.

## Figures and Tables

**Table 1:** Structural characteristics of the mitogenome of *P. grumi*.

Gene	Position		Size/bp	Intergenic Nucleotides	Codon		Strand
	From	To			Start	Stop	
*tRNA-Phe*	1	69	69	-	-	-	H
*12S rRNA*	70	1024	955	-	-	-	H
*tRNA-Val*	1025	1096	72	-	-	-	H
*16S rRNA*	1097	2786	1690	-	-	-	H
*tRNA-Leu1*	2787	2862	76	-	-	-	H
*ND1*	2864	3838	975	1	ATG	TAA	H
*tRNA-Ile*	3842	3913	72	3	-	-	H
*tRNA-Gln*	3912	3982	71	−2	-	-	L
*tRNA-Met*	3984	4052	69	1	-	-	H
*ND2*	4053	5097	1045	-	ATG	T-	H
*tRNA-Trp*	5098	5168	71	-	-	-	H
*tRNA-Ala*	5170	5238	69	1	-	-	L
*tRNA-Asn*	5240	5312	73	1	-	-	L
*tRNA-Cys*	5345	5412	68	32	-	-	L
*tRNA-Tyr*	5414	5484	71	1	-	-	L
*COX1*	5486	7036	1551	1	GTG	TAA	H
*tRNA-Ser1*	7037	7107	71	-	-	-	L
*tRNA-Asp*	7110	7183	74	2	-	-	H
*COX2*	7197	7887	691	13	ATG	T-	H
*tRNA-Lys*	7888	7963	76	-	-	-	H
*ATP8*	7965	8129	165	1	ATG	TAA	H
*ATP6*	8123	8805	683	−7	ATG	TA-	H
*COX3*	8806	9589	784	-	ATG	T-	H
*tRNA-Gly*	9590	9660	71	-	-	-	H
*ND3*	9661	10,009	349	-	ATG	T-	H
*tRNA-Arg*	10,010	10,078	69	-	-	-	H
*ND4L*	10,079	10,375	297	-	ATG	TAA	H
*ND4*	10,369	11,750	1382	−7	ATG	TA-	H
*tRNA-His*	11,751	11,819	69	-	-	-	H
*tRNA-Ser2*	11,820	11,887	68	-	-	-	H
*tRNA-Leu2*	11,889	11,961	73	1	-	-	H
*ND5*	11,962	13,797	1836	-	ATG	TAA	H
*ND6*	13,794	14,315	522	−4	ATG	TAA	L
*tRNA-Glu*	14,316	14,384	69	-	-	-	L
*Cytb*	14,389	15,529	1141	4	ATG	T-	H
*tRNA-Thr*	15,530	15,601	72	-	-	-	H
*tRNA-Pro*	15,601	15,670	70	−1	-	-	L
D-loop	15,671	16,604	933	-	-	-	-

Note: H represents the heavy strand; L represents the light strand. “–” indicates that the item is not applicable.

**Table 2:** Composition and skewness of the *P. grumi* mitogenome.

Regions	Size (bp)	T (U)	C	A	G	AT (%)	GC (%)	AT Skew	GC Skew
Protein-coding genes	11,412	29.5	27.1	25.3	18.1	54.8	45.2	−0.077	−0.201
1st codon position	3804	21.6	25.6	25.9	26.9	47.5	52.5	0.091	0.025
2nd codon position	3804	40.5	27.6	18	13.9	58.5	41.5	−0.385	−0.33
3rd codon position	3804	26.5	28.2	32	13.4	58.5	41.6	0.094	−0.357
*ATP6*	683	30	28	26.5	15.5	56.5	43.5	−0.062	−0.286
*ATP8*	165	24.8	27.9	34.5	12.7	59.3	40.6	0.163	−0.373
*COX1*	1551	31.7	24.4	24.9	19	56.6	43.4	−0.121	−0.123
*COX2*	691	28.8	24.6	28.7	17.9	57.5	42.5	−0.003	−0.156
*COX3*	784	30.2	26.7	24	19.1	54.2	45.8	−0.115	−0.164
*Cytb*	1141	31.2	27	25.9	16	57.1	43	−0.094	−0.257
*ND1*	975	28	29.8	23.2	19	51.2	48.8	−0.094	−0.223
*ND2*	1045	26.3	31.6	25	17.1	51.3	48.7	−0.026	−0.297
*ND3*	349	29.8	28.1	22.9	19.2	52.7	47.3	−0.13	−0.188
*ND4*	1382	28.8	28	26	17.2	54.8	45.2	−0.052	−0.238
*ND4L*	297	28.3	29.6	25.3	16.8	53.6	46.4	−0.057	−0.275
*ND5*	1836	28.5	28.5	27.3	15.7	55.8	44.2	−0.021	−0.288
*ND6*	522	36.2	14.9	15.3	33.5	51.5	48.4	−0.405	0.383
16S rRNA	1690	21.1	23.4	33.3	22.3	54.4	45.7	0.224	−0.023
12S rRNA	955	20.3	25.5	29.8	24.3	50.1	49.8	0.19	−0.025
rRNAs	2645	20.8	24.2	32	23	52.8	47.2	0.213	−0.024
tRNAs	1563	26	21.8	28.5	23.7	54.5	45.5	0.046	0.042
Full genome	16,604	27.2	26.7	27.7	18.4	54.9	45.1	0.008	−0.184

## Data Availability

The complete mitochondrial genome of *P. grumi* has been deposited in the GenBank public database (http://www.ncbi.nlm.nih.gov) under the accession number PZ250837.1.

## Supplementary Materials

The following supporting information can be downloaded at https://www.mdpi.com/article/10.3390/genes17060635/s1, Table S1: The list of mitochondrial genomes from Leuciscidae species used for phylogenetic analysis, with Cobitis striata and Cyprinus carpio designated as the outgroup; Table S2: Genetic distance matrix analysis of 31 species based on concatenated sequences of 13 PCGs; Figure S1: Maximum likelihood (ML) phylogenetic tree of Leuciscidae constructed based on the nucleotide sequences of the mitochondrial *Cytb* gene; Figure S2: Bayesian inference (BI) phylogenetic tree of Leuciscidae constructed based on the nucleotide sequences of the mitochondrial *Cytb* gene.

## References

[1] Betancur-R R., Wiley E.O., Arratia G., Acero A., Bailly N., Miya M., Lecointre G., Ortí G. (2017). Phylogenetic classification of bony fishes. BMC Evol. Biol..

[2] Mayden R.L., Chen W.J., Bart H.L., Doosey M.H., Simons A.M., Tang K.L., Wood R.M., Agnew M.K., Yang L., Hirt M.V. (2009). Reconstructing the phylogenetic relationships of the earth’s most diverse clade of freshwater fishes–order Cypriniformes (Actinopterygii: Ostariophysi): A case study using multiple nuclear loci and the mitochondrial genome. Mol. Phylogenet. Evol..

[3] Hao C., Liu Y., Wei N., Arken K., Shi C., Yue C. (2023). The complete mitochondrial genomes of the Leuciscus baicalensis and Rutilus rutilus: A detailed genomic comparison among closely related species of the Leuciscinae subfamily. Gene.

[4] Bogutskaya N.G., Naseka A.M. (2004). Catalogue of Agnathans and Fishes of Fresh and Brackish Waters of Russia with Comments on Nomenclature and Taxonomy.

[5] Kubanç N., Eldem V., Kubanç C. (2016). The complete mitochondrial genome of *Alburnus tarichi* (Teleostei, Cyprinidae). Mitochondrial DNA Part A DNA Mapp. Seq. Anal..

[6] Schönhuth S., Vukić J., Šanda R., Yang L., Mayden R.L. (2018). Phylogenetic relationships and classification of the Holarctic family Leuciscidae (Cypriniformes: Cyprinoidei). Mol. Phylogenet. Evol..

[7] Tang K.L., Agnew M.K., Hirt M.V., Sado T., Schneider L.M., Freyhof J., Sulaiman Z., Swartz E., Vidthayanon C., Miya M. (2010). Systematics of the subfamily Danioninae (Teleostei: Cypriniformes: Cyprinidae). Mol. Phylogenet. Evol..

[8] Miya M., Nishida M. (2015). The mitogenomic contributions to molecular phylogenetics and evolution of fishes: A 15-year retrospect. Ichthyol. Res..

[9] DeSalle R., Tessler M. (2025). Mitochondrial Gene Phylogenetic Incongruencies Are Linked to Chromosomal Position and Function. Genome Biol. Evol..

[10] Perea S., Böhme M., Zupancic P., Freyhof J., Šanda R., Özuluğ M., Abdoli A., Doadrio I. (2010). Phylogenetic relationships and biogeographical patterns in Circum-Mediterranean subfamily Leuciscinae (Teleostei, Cyprinidae) inferred from both mitochondrial and nuclear data. BMC Evol. Biol..

[11] Imoto J.M., Saitoh K., Sasaki T., Yonezawa T., Adachi J., Kartavtsev Y.P., Miya M., Nishida M., Hanzawa N. (2013). Phylogeny and biogeography of highly diverged freshwater fish species (Leuciscinae, Cyprinidae, Teleostei) inferred from mitochondrial genome analysis. Gene.

[12] Boore J.L. (1999). Animal mitochondrial genomes. Nucleic Acids Res..

[13] Wolstenholme D.R. (1992). Animal mitochondrial DNA: Structure and evolution. Int. Rev. Cytol..

[14] Saccone C., De Giorgi C., Gissi C., Pesole G., Reyes A. (1999). Evolutionary genomics in Metazoa: The mitochondrial DNA as a model system. Gene.

[15] Saitoh K., Sado T., Mayden R.L., Hanzawa N., Nakamura K., Nishida M., Miya M. (2006). Mitogenomic evolution and interrelationships of the Cypriniformes (Actinopterygii: Ostariophysi): The first evidence toward resolution of higher-level relationships of the world’s largest freshwater fish clade based on 59 whole mitogenome sequences. J. Mol. Evol..

[16] Zhang C., Zhao Y. (2016). Species Diversity and Distribution of Inland Fishes in China.

[17] Chen Y.Y. (1998). Fauna Sinica: Osteichthyes: Cypriniformes (Volume II).

[18] Cao L., Shao W.H., Yi W.J., Zhang E. (2024). A review of conservation status of freshwater fish diversity in China. J. Fish Biol..

[19] Du L., Wong J.S., Li Z., Chen L., Zhang B., Lei B., Peng Z. (2023). Hydroclimatic Change in Turpan Basin under Climate Change. Water.

[20] Guo Y., Zhang R.M., Cai L.G. (2012). Fishes of Xinjiang.

[21] Wang J., Chang H., Yang P., Wang X., Li X., He Y., Gao M., Guo W. (2026). A chromosomal-level genome assembly of *Phoxinus grumi* (Cypriniformes: Leuciscidae). Sci. Data.

[22] Strange R.M., Mayden R.L. (2009). Phylogenetic Relationships and a Revised Taxonomy for North American Cyprinids Currently Assigned to *Phoxinus* (Actinopterygii: Cyprinidae). Copeia.

[23] Ito Y., Sakai H., Shedko S., Jeon S.R. (2002). Genetic differentiation of the northern Far East cyprinids, Phoxinus and Rhynchocypris. Fish. Sci..

[24] Sakai H., Ito Y., Shedko S.V., Safronov S.N., Frolov S.V., Chereshnev I.A., Jeon S.R., Goto A. (2006). Phylogenetic and taxonomic relationships of northern Far Eastern phoxinin minnows, *Phoxinus* and *Rhynchocypris* (Pisces, Cyprinidae), as inferred from allozyme and mitochondrial 16S rRNA sequence analyses. Zool. Sci..

[25] Zardoya R., Doadrio I. (1999). Molecular evidence on the evolutionary and biogeographical patterns of European cyprinids. J. Mol. Evol..

[26] Arai R. (1982). A chromosome study on two Cyprinid Fishes, *Acrossocheilus labiatus* and *Pseudorasbora pumila pumila*, with notes on Eurasian Cyprinids and their karyotypes. Bull. Natl. Sci. Mus..

[27] Kawamura K. (2008). “Handbook of European freshwater fishes” by M. Kottelat and J. Freyhof (2007). Ichthyol. Res..

[28] Chen X.L., Yue P.Q., Lin R.D. (1984). Major groups within the family Cyprinidae and their phylogenetic relationship. Acta Zootax. Sin..

[29] Simons A.M., Mayden R.L. (1999). Phylogenetic Relationships of North American Cyprinids and Assessment of Homology of the Open Posterior Myodome. Copeia.

[30] Museth J., Hesthagen T., Sandlund O.T., Thorstad E.B., Ugedal O. (2007). The history of the minnow *Phoxinus phoxinus* (L.) in Norway: From harmless species to pest. J. Fish Biol..

[31] Chen S., Zhou Y., Chen Y., Gu J. (2018). fastp: An ultra-fast all-in-one FASTQ preprocessor. Bioinformatics.

[32] Jin J.J., Yu W.B., Yang J.B., Song Y., dePamphilis C.W., Yi T.S., Li D.Z. (2020). GetOrganelle: A fast and versatile toolkit for accurate de novo assembly of organelle genomes. Genome Biol..

[33] Shen W., Le S., Li Y., Hu F. (2016). SeqKit: A Cross-Platform and Ultrafast Toolkit for FASTA/Q File Manipulation. PLoS ONE.

[34] Wick R.R., Schultz M.B., Zobel J., Holt K.E. (2015). Bandage: Interactive visualization of de novo genome assemblies. Bioinformatics.

[35] Camacho C., Coulouris G., Avagyan V., Ma N., Papadopoulos J., Bealer K., Madden T.L. (2009). BLAST+: Architecture and applications. BMC Bioinform..

[36] Zheng S., Poczai P., Hyvönen J., Tang J., Amiryousefi A. (2020). Chloroplot: An Online Program for the Versatile Plotting of Organelle Genomes. Front. Genet..

[37] Bernt M., Donath A., Jühling F., Externbrink F., Florentz C., Fritzsch G., Pütz J., Middendorf M., Stadler P.F. (2013). MITOS: Improved de novo metazoan mitochondrial genome annotation. Mol. Phylogenet. Evol..

[38] Iwasaki W., Fukunaga T., Isagozawa R., Yamada K., Maeda Y., Satoh T.P., Sado T., Mabuchi K., Takeshima H., Miya M. (2013). MitoFish and MitoAnnotator: A mitochondrial genome database of fish with an accurate and automatic annotation pipeline. Mol. Biol. Evol..

[39] Lowe T.M., Chan P.P. (2016). tRNAscan-SE On-line: Integrating search and context for analysis of transfer RNA genes. Nucleic Acids Res..

[40] Kearse M., Moir R., Wilson A., Stones-Havas S., Cheung M., Sturrock S., Buxton S., Cooper A., Markowitz S., Duran C. (2012). Geneious Basic: An integrated and extendable desktop software platform for the organization and analysis of sequence data. Bioinformatics.

[41] Zhang D., Gao F., Jakovlić I., Zou H., Zhang J., Li W.X., Wang G.T. (2020). PhyloSuite: An integrated and scalable desktop platform for streamlined molecular sequence data management and evolutionary phylogenetics studies. Mol. Ecol. Resour..

[42] Yang Z., Nielsen R. (2000). Estimating synonymous and nonsynonymous substitution rates under realistic evolutionary models. Mol. Biol. Evol..

[43] Wang D., Zhang Y., Zhang Z., Zhu J., Yu J. (2010). KaKs_Calculator 2.0: A toolkit incorporating gamma-series methods and sliding window strategies. Genom. Proteom. Bioinform..

[44] Zhang Y.T., Lu C.P., Tao Y.H., Tong C.W., Chen J., Liu W. (2025). The complete mitochondrial genome of *Rhynchocypris czekanowskii* (Cypriniformes, Cyprinidae). Mitochondrial DNA Part B Resour..

[45] Nguyen L.T., Schmidt H.A., von Haeseler A., Minh B.Q. (2015). IQ-TREE: A fast and effective stochastic algorithm for estimating maximum-likelihood phylogenies. Mol. Biol. Evol..

[46] Katoh K., Standley D.M. (2013). MAFFT multiple sequence alignment software version 7: Improvements in performance and usability. Mol. Biol. Evol..

[47] Kalyaanamoorthy S., Minh B.Q., Wong T.K.F., von Haeseler A., Jermiin L.S. (2017). ModelFinder: Fast model selection for accurate phylogenetic estimates. Nat. Methods.

[48] Ronquist F., Teslenko M., van der Mark P., Ayres D.L., Darling A., Höhna S., Larget B., Liu L., Suchard M.A., Huelsenbeck J.P. (2012). MrBayes 3.2: Efficient Bayesian phylogenetic inference and model choice across a large model space. Syst. Biol..

[49] Rambaut A., Drummond A.J., Xie D., Baele G., Suchard M.A. (2018). Posterior Summarization in Bayesian Phylogenetics Using Tracer 1.7. Syst. Biol..

[50] Letunic I., Bork P. (2024). Interactive Tree of Life (iTOL) v6: Recent updates to the phylogenetic tree display and annotation tool. Nucleic Acids Res..

[51] Kimura M. (1980). A simple method for estimating evolutionary rates of base substitutions through comparative studies of nucleotide sequences. J. Mol. Evol..

[52] Lee Y.J., Cha S.H., An J., Suk H.Y. (2019). The complete mitochondrial genome information of *Phoxinus phoxinus* (Cypriniformes: Cyprinidae) on the Korean Peninsula and the phylogenetic implication. Mitochondrial DNA Part B Resour..

[53] Xu W., Chen A., Xia R., Fu C. (2014). Complete mitochondrial genome of *Rhynchocypris* cf. *lagowskii* (Cypriniformes: Cyprinidae). Mitochondrial DNA.

[54] Wang T., Song F., Yang Z., Su Z., Shi X., Zhang Y., Lv B., Du Y. (2025). The complete mitochondrial genome of *Leuciscus chuanchicus* (Kessler, 1876) (Cypriniformes: Cyprinidae). Mitochondrial DNA Part B Resour..

[55] Qi D., Chao Y., Zhao L., Shen Z., Wang G. (2013). Complete mitochondrial genomes of two relatively closed species from *Gymnocypris* (Cypriniformes: Cyprinidae): Genome characterization and phylogenetic considerations. Mitochondrial DNA.

[56] Chen W.F., Peng X., Yu Y., Yang C.M., Chen X., Zhao J., Wang J. (2019). Complete mitochondrial genome and the phylogenetic position of the stellate puffer (*Arothron stellatus*). Mitochondrial DNA Part B Resour..

[57] Haÿ V., Pividori R., Dettai A., Delling B., Denys G.P.J. (2025). The complete mitochondrial genome of the common dace *Leuciscus leuciscus* (Teleostei: Leuciscidae). Mitochondrial DNA Part B Resour..

[58] Takahashi H., Møller P.R., Shedko S.V., Ramatulla T., Joen S.R., Zhang C.G., Sideleva V.G., Takata K., Sakai H., Goto A. (2016). Species phylogeny and diversification process of Northeast Asian Pungitius revealed by AFLP and mtDNA markers. Mol. Phylogenet. Evol..

[59] Xie P., Ao M., Liu C., Zhang Z., Zhang Y., Niu J., Karjan A., Ma X. (2016). The complete mitochondrial genome of *Phoxinus phoxinus ujmonensis* (Cypriniformes: Cyprinidae). Mitochondrial DNA Part A DNA Mapp. Seq. Anal..

[60] Satoh T.P., Miya M., Mabuchi K., Nishida M. (2016). Structure and variation of the mitochondrial genome of fishes. BMC Genom..

[61] Xu W., Geng L.W., Xu M., Tong G.X., Jiang H.F. (2016). Mitochondrial DNA sequence of *Pseudorasbora parva* (Cyprinidae: Gobioninae). Mitochondrial DNA Part A DNA Mapp. Seq. Anal..

[62] Ojala D., Montoya J., Attardi G. (1981). tRNA punctuation model of RNA processing in human mitochondria. Nature.

[63] Cheng L., Wang E., Li W., Yu X., Liao X. (2022). The Complete Mitochondrial Genome of Eurasian Minnow (*Phoxinus* cf. *phoxinus*) from the Heilongjiang River, and Its Phylogenetic Implications. Animals.

[64] Zhang Z., Li J., Zhang X., Lin B., Chen J. (2022). Comparative mitogenomes provide new insights into phylogeny and taxonomy of the subfamily *Xenocyprinae* (Cypriniformes: Cyprinidae). Front. Genet..

[65] Zhang R., Zhu T., Luo Q. (2023). The Complete Mitochondrial Genome of the Freshwater Fish *Onychostoma ovale* (Cypriniformes, Cyprinidae): Genome Characterization and Phylogenetic Analysis. Genes.

[66] Nekrutenko A., Makova K.D., Li W.H. (2002). The K(A)/K(S) ratio test for assessing the protein-coding potential of genomic regions: An empirical and simulation study. Genome Res..

[67] Zhang Z., Cheng Q., Ge Y. (2019). The complete mitochondrial genome of *Rhynchocypris oxycephalus* (Teleostei: Cyprinidae) and its phylogenetic implications. Ecol. Evol..

[68] Froufe E., Knizhin I., Weiss S. (2005). Phylogenetic analysis of the genus *Thymallus* (grayling) based on mtDNA control region and ATPase 6 genes, with inferences on control region constraints and broad-scale Eurasian phylogeography. Mol. Phylogenet. Evol..

[69] Phillips R.B., Oakley T.H., Kocher T.D. (1997). Phylogenetic Relationships among the Salmoninae Based on Nuclear and Mitochondrial DNA Sequences. Molecular Systematics of Fishes.

[70] Sousa-Santos C., Jesus T.F., Fernandes C., Robalo J.I., Coelho M.M. (2019). Fish diversification at the pace of geomorphological changes: Evolutionary history of western Iberian Leuciscinae (Teleostei: Leuciscidae) inferred from multilocus sequence data. Mol. Phylogenet. Evol..

[71] Sun M., Soltis D.E., Soltis P.S., Zhu X., Burleigh J.G., Chen Z. (2015). Deep phylogenetic incongruence in the angiosperm clade Rosidae. Mol. Phylogenet. Evol..

[72] Song N., Liang A.P., Bu C.P. (2012). A molecular phylogeny of Hemiptera inferred from mitochondrial genome sequences. PLoS ONE.

[73] Kusznierz J., Tagayev D., Sienkiewicz T., Paśko Ł. (2023). Molecular and osteological verification of the taxonomic status of *Phoxinus sedelnikowi* (Berg, 1908) (Teleostei: Leuciscidae). Eur. Zool. J..

[74] Sakai H., Watanabe K., Goto A. (2020). A revised generic taxonomy for Far East Asian minnow *Rhynchocypris* and dace *Pseudaspius*. Ichthyol. Res..

