# Supplementary material for: Complete Mitochondrial Genome of Phoxinus grumi (Cypriniformes: Leuciscidae): Characterization and Phylogenetic Position
Source: Genes (Basel). 2026 May 30;17(6):635. doi: 10.3390/genes17060635 (PMC13299415; doi:10.3390/genes17060635)
Supplement: Supplementary file 1 [file genes-17-00635-s001.zip › Figure S1 and S2.pdf]

(S1)

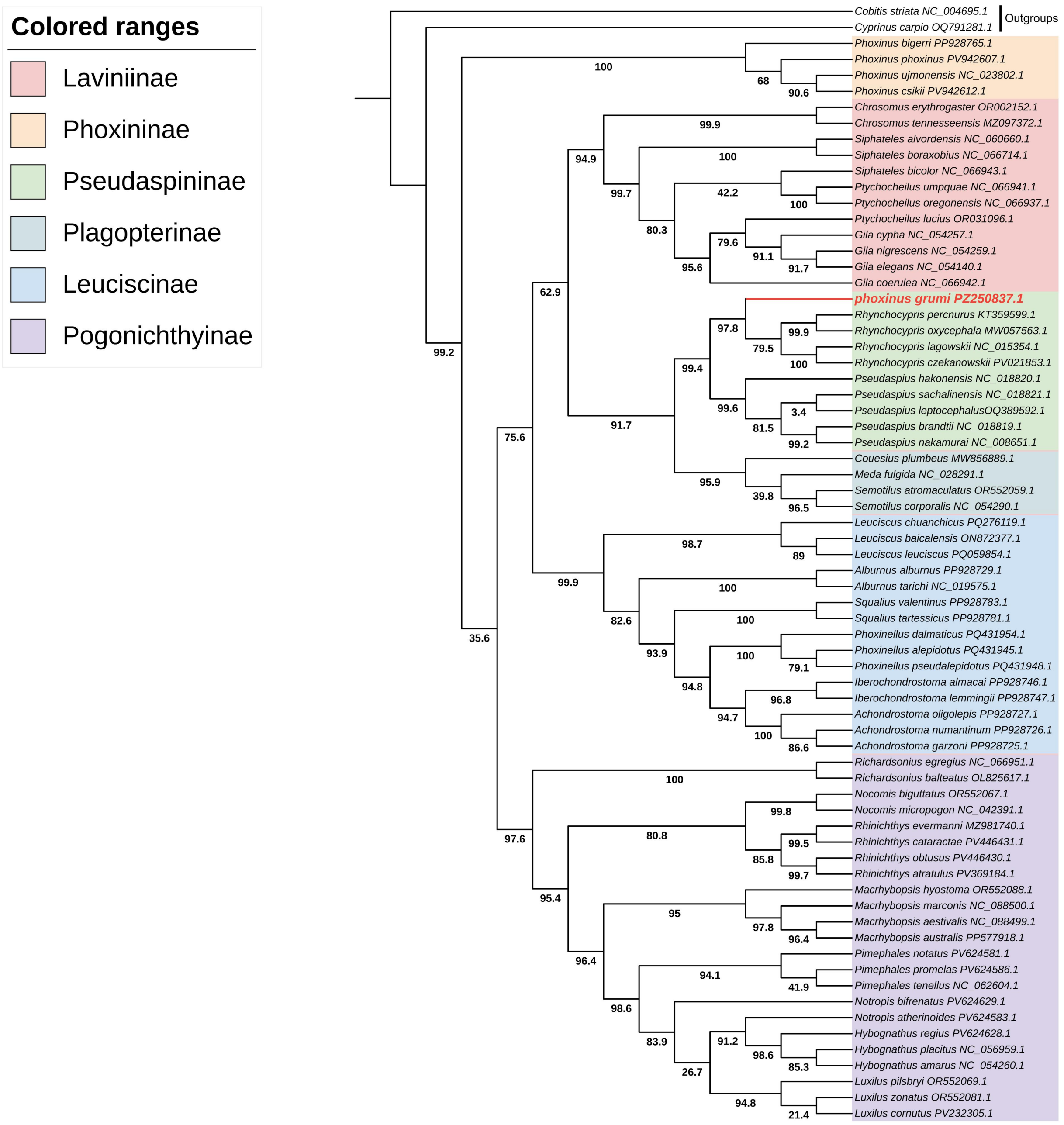

Figure S1. Maximum likelihood (ML) phylogenetic tree of Leuciscidae constructed based on the nucleotide sequences of the mitochondrial *Cytb* gene. ML tree constructed using IQ-TREE, where bootstrap value ≥ 70 indicates high support, 50 ≤ bootstrap value < 70 indicates medium support, and bootstrap value < 50 indicates low support. The numbers below the branches represent confidence levels.

(S2)

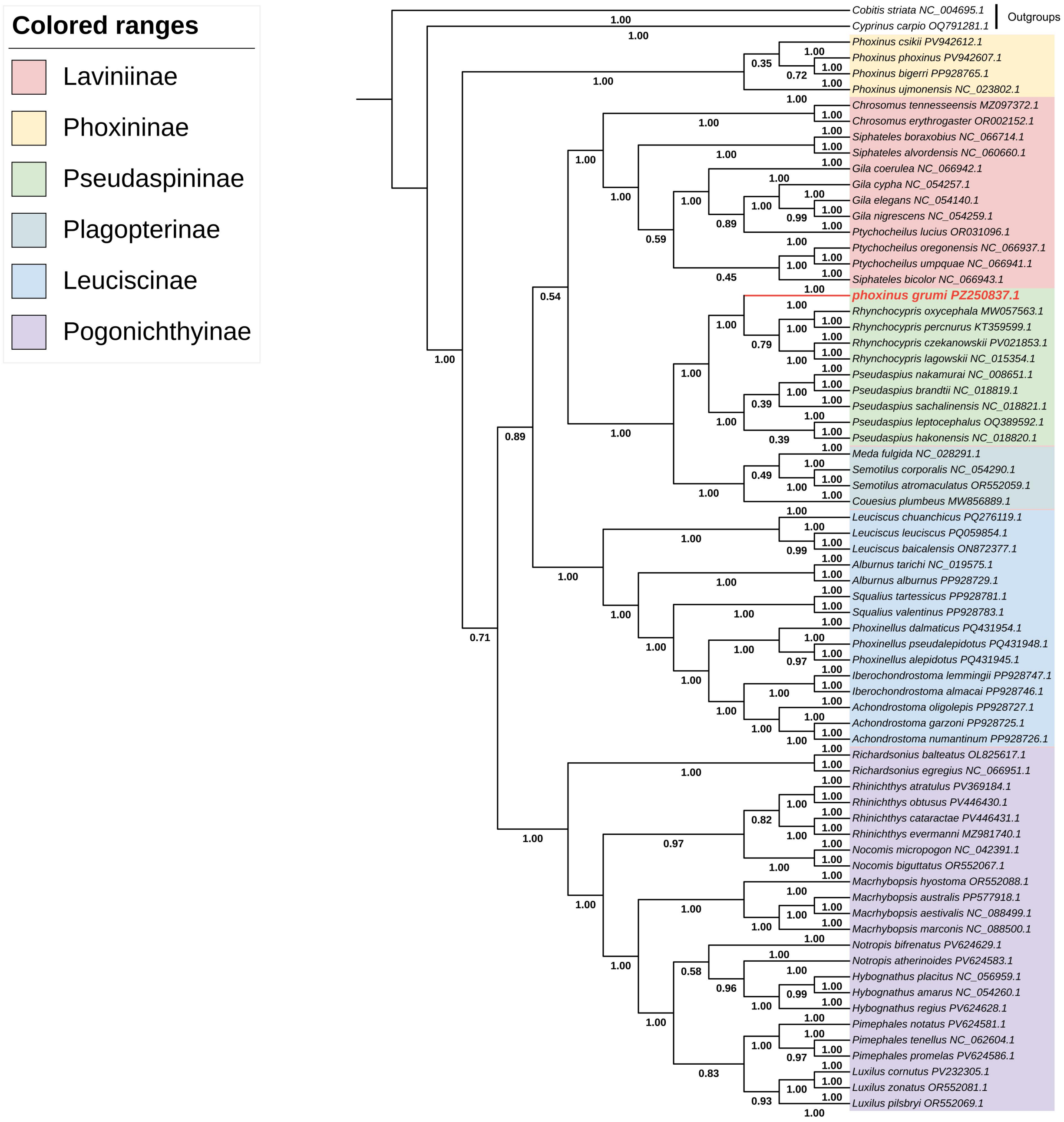

Figure S2. Bayesian inference (BI) phylogenetic tree of Leuciscidae constructed based on the nucleotide sequences of the mitochondrial *Cytb* gene. BI tree constructed using MrBayes, where posterior probability (PP) $\geq$ 0.95 indicates high node support, 0.70 $\leq$ PP < 0.95 indicates medium node support, and PP < 0.70 indicates low node support. The numbers below the branches represent confidence levels.
