# Supplementary material for: Complete Mitochondrial Genome of Phoxinus grumi (Cypriniformes: Leuciscidae): Characterization and Phylogenetic Position
Source: Genes (Basel). 2026 May 30;17(6):635. doi: 10.3390/genes17060635 (PMC13299415; doi:10.3390/genes17060635)
Supplement: Supplementary file 1 [file genes-17-00635-s001.zip › Table S1.pdf]

Table S1. A list of mitochondrial genomes from Leuciscidae species were used for phylogenetic analysis, with *Cobitis striata* and *Cyprinus carpio* designated as outgroup.

Subfamily	Genus	Species	Length(bp)	GenBank Accession Number
Laviniinae	Chrosomus	Chrosomus tennesseensis	16596 bp	MZ097372.1
		Chrosomus erythrogaster	16599 bp	OR002152.1
	Gila	Gila coerulea	16589 bp	NC_066942.1
		Gila elegans	16593 bp	NC_054140.1
		Gila nigrescens	16585 bp	NC_054259.1
		Gila cypha	16591 bp	NC_054257.1
	Ptychocheilus	Ptychocheilus lucius	16588 bp	OR031096.1
		Ptychocheilus umpquae	16605 bp	NC_066941.1
		Ptychocheilus oregonensis	16605 bp	NC_066937.1
	Siphateles	Siphateles boraxobius	16599 bp	NC_066714.1
		Siphateles bicolor	16602 bp	NC_066943.1
		Siphateles alvordensis	16599 bp	NC_060660.1
Leuciscinae	Achondrostoma	Achondrostoma oligolepis	16602 bp	PP928727.1
		Achondrostoma numantinum	16602 bp	PP928726.1
		Achondrostoma garzoni	16602 bp	PP928725.1
	Alburnus	Alburnus alburnus	16605 bp	PP928729.1
		Alburnus tarichi	16602 bp	NC_019575.1
	Iberochondrostoma	Iberochondrostoma lemmingii	16601 bp	PP928747.1
		Iberochondrostoma almacai	16604 bp	PP928746.1
	Leuciscus	Leuciscus chuanchicus	16602 bp	PQ276119.1
		Leuciscus leuciscus	16603 bp	PQ059854.1
		Leuciscus baicalensis	16607 bp	ON872377.1
	Phoxinellus	Phoxinellus dalmaticus	16600 bp	PQ431954.1
		Phoxinellus pseudalepidotus	16600 bp	PQ431948.1
		Phoxinellus alepidotus	16601 bp	PQ431945.1
	Squalius	Squalius valentinus	16606 bp	PP928783.1
		Squalius tartessicus	16607 bp	PP928781.1
Phoxininae	Phoxinus	Phoxinus csikii	16605 bp	PV942612.1
		Phoxinus phoxinus	16671 bp	PV942607.1
		Phoxinus bigerri	17536 bp	PP928765.1
		Phoxinus ujmonensis	17738 bp	NC_023802.1
Plagopterinae	Couesius	Couesius plumbeus	16604 bp	MW856889.1
	Meda	Meda fulgida	16591 bp	NC_028291.1
	Semotilus	Semotilus atromaculatus	16622 bp	OR552059.1
		Semotilus corporalis	16602 bp	NC_054290.1
Pogonichthyinae	Hybognathus	Hybognathus regius	16711 bp	PV624628.1
		Hybognathus placitus	16709 bp	NC_056959.1
		Hybognathus amarus	16714 bp	NC_054260.1
	Luxilus	Luxilus cornutus	16717 bp	PV232305.1
		Luxilus zonatus	16716 bp	OR552081.1
		Luxilus pilsbryi	16680 bp	OR552069.1

Subfamily	Genus	Species	Length(bp)	GenBank Accession Number
	Macrhybopsis	Macrhybopsis marconis	16702 bp	NC_088500.1
		Macrhybopsis aestivalis	16645 bp	NC_088499.1
		Macrhybopsis australis	16713 bp	PP577918.1
		Macrhybopsis hyostoma	16846 bp	OR552088.1
	Nocomis	Nocomis biguttatus	16656 bp	OR552067.1
		Nocomis micropogon	16655 bp	NC_042391.1
	Notropis	Notropis bifrenatus	16709 bp	PV624629.1
		Notropis atherinoides	16710 bp	PV624583.1
	Pimephales	Pimephales promelas	16709 bp	PV624586.1
		Pimephales notatus	16706 bp	PV624581.1
		Pimephales tenellus	16710 bp	NC_062604.1
	Rhinichthys	Rhinichthys cataractae	16659 bp	PV446431.1
		Rhinichthys obtusus	16650 bp	PV446430.1
		Rhinichthys atratulus	16646 bp	PV369184.1
		Rhinichthys evermanni	16658 bp	MZ981740.1
	Richardsonius	Richardsonius egregius	16646 bp	NC_066951.1
		Richardsonius balteatus	16647 bp	OL825617.1
Pseudaspininae	Pseudaspius	Pseudaspius leptocephalus	16607 bp	OQ389592.1
		Pseudaspius hakonensis	16602 bp	NC_018820.1
		Pseudaspius sachalinensis	16597 bp	NC_018821.1
		Pseudaspius brandtii	16598 bp	NC_018819.1
		Pseudaspius nakamurai	16616 bp	NC_008651.1
	Rhynchocypris	Rhynchocypris czekanowskii	16651 bp	PV021853.1
		Rhynchocypris oxycephala	16608 bp	MW057563.1
		Rhynchocypris percnurus	16608 bp	KT359599.1
		Rhynchocypris lagowskii	16599 bp	NC_015354.1
Cobitidae	Cobitis	Cobitis striata	16572 bp	NC_004695.1
Cyprininae	Cyprinus	Cyprinus carpio	16581 bp	OQ791281.1
