# Supplementary material for: Complete Mitochondrial Genome of Phoxinus grumi (Cypriniformes: Leuciscidae): Characterization and Phylogenetic Position
Source: Genes (Basel). 2026 May 30;17(6):635. doi: 10.3390/genes17060635 (PMC13299415; doi:10.3390/genes17060635)
Supplement: Supplementary file 1 [file genes-17-00635-s001.zip › Table S2.pdf]

Table S2 Genetic distance matrix analysis of 31 species based on concatenated sequences of 13 PCGs

			A	B	C	D	E	F	G	H	I	J	K	L	M	N	O	P	Q	R	S	T	U	V	W	X	Y	Z	AA	AB	AC	AD
Plagopterinae	*phoxinus grumi*	A																														
	Rhynchocypris czekanowskii	B	0.1620																													
	Rhynchocypris lagowskii	C	0.1628	0.0103																												
	Rhynchocypris oxycephala	D	0.1703	0.1219	0.1242																											
	Rhynchocypris percnurus	E	0.1703	0.1219	0.1242	0.0000																										
	Pseudaspius brandtii	F	0.1832	0.1608	0.1611	0.1647	0.1647																									
	Pseudaspius hakonensis	G	0.1853	0.1633	0.1636	0.1689	0.1689	0.0964																								
	Pseudaspius leptocephalus	H	0.1857	0.1641	0.1660	0.1688	0.1688	0.0792	0.0984																							
	Pseudaspius nakamurai	I	0.1852	0.1633	0.1634	0.1650	0.1650	0.0483	0.1011	0.0849																						
	Pseudaspius sachalinensis	J	0.1843	0.1609	0.1611	0.1665	0.1665	0.0831	0.0936	0.0833	0.0848																					
Phoxininae	*Phoxinus bigerri*	K	0.2505	0.2389	0.2324	0.2429	0.2429	0.2399	0.2437	0.2492	0.2403	0.2423																				
	Phoxinus csikii	L	0.2475	0.2348	0.2299	0.2418	0.2418	0.2313	0.2367	0.2405	0.2327	0.2327	0.0837																			
	Phoxinus phoxinus	M	0.2558	0.2380	0.2321	0.2394	0.2394	0.2410	0.2397	0.2413	0.2382	0.2368	0.0780	0.0838																		
	Phoxinus ujmonensis	N	0.2539	0.2375	0.2305	0.2429	0.2429	0.2372	0.2460	0.2469	0.2410	0.2385	0.0886	0.0878	0.0959																	
Laviniinae	*Chrosomus erythrogaster*	O	0.2298	0.2115	0.2128	0.2190	0.2190	0.2040	0.2055	0.2119	0.2062	0.2063	0.2343	0.2317	0.2372	0.2324																
	Chrosomus tennesseensis	P	0.2280	0.2208	0.2199	0.2169	0.2169	0.2052	0.2086	0.2182	0.2092	0.2102	0.2395	0.2350	0.2350	0.2384	0.1309															
	Gila coerulea	Q	0.2264	0.2046	0.2052	0.2081	0.2081	0.1995	0.1997	0.2059	0.2026	0.2016	0.2268	0.2231	0.2251	0.2256	0.1705	0.1759														
	Gila cypha	R	0.2222	0.2043	0.2040	0.2064	0.2064	0.1954	0.1947	0.2074	0.1978	0.1983	0.2296	0.2189	0.2259	0.2240	0.1725	0.1735	0.0645													
	Gila elegans	S	0.2265	0.2108	0.2102	0.2108	0.2108	0.1984	0.2024	0.2081	0.2030	0.2026	0.2285	0.2244	0.2262	0.2280	0.1775	0.1775	0.0694	0.0571												
	Gila nigrescens	T	0.2242	0.2065	0.2042	0.2097	0.2097	0.1974	0.1962	0.2052	0.1998	0.1990	0.2281	0.2208	0.2256	0.2256	0.1696	0.1785	0.0677	0.0546	0.0597											
	Ptychocheilus lucius	U	0.2227	0.2024	0.1998	0.2065	0.2065	0.1965	0.1987	0.2059	0.2005	0.1988	0.2235	0.2191	0.2221	0.2230	0.1674	0.1747	0.0653	0.0573	0.0644	0.0631										
	Ptychocheilus umpquae	V	0.2219	0.2028	0.2029	0.2072	0.2072	0.1992	0.1971	0.2026	0.1990	0.1949	0.2221	0.2191	0.2204	0.2218	0.1676	0.1768	0.0811	0.0826	0.0897	0.0865	0.0827									
	Ptychocheilus oregonensis	W	0.2216	0.1992	0.2001	0.2047	0.2047	0.1974	0.1948	0.2046	0.1974	0.1950	0.2219	0.2168	0.2201	0.2202	0.1678	0.1755	0.0782	0.0776	0.0869	0.0820	0.0791	0.0194								
	Siphateles alvordensis	X	0.2291	0.2074	0.2058	0.2107	0.2107	0.2021	0.2072	0.2096	0.2044	0.2072	0.2274	0.2286	0.2277	0.2301	0.1871	0.1956	0.1186	0.1174	0.1270	0.1218	0.1213	0.1149	0.1153							
	Siphateles bicolor	Y	0.2278	0.2052	0.2062	0.2087	0.2087	0.1966	0.1986	0.2055	0.2005	0.1991	0.2248	0.2202	0.2233	0.2240	0.1680	0.1766	0.0880	0.0861	0.0929	0.0915	0.0879	0.0825	0.0803	0.1193						
	Siphateles boraxobius	Z	0.2299	0.2076	0.2060	0.2104	0.2104	0.2024	0.2070	0.2098	0.2046	0.2069	0.2279	0.2291	0.2282	0.2312	0.1868	0.1959	0.1186	0.1174	0.1277	0.1218	0.1213	0.1150	0.1154	0.0009	0.1193					
Plagopterinae	*Couesius plumbeus*	AA	0.2341	0.2080	0.2077	0.2085	0.2085	0.1974	0.1987	0.2035	0.2004	0.1954	0.2360	0.2359	0.2356	0.2399	0.2046	0.2145	0.1915	0.1919	0.1925	0.1876	0.1891	0.1913	0.1892	0.1994	0.1906	0.1998				
	Semotilus atromaculatus	AB	0.2372	0.2191	0.2184	0.2213	0.2213	0.2116	0.2183	0.2220	0.2174	0.2208	0.2513	0.2441	0.2428	0.2426	0.2156	0.2243	0.2128	0.2101	0.2122	0.2136	0.2108	0.2095	0.2078	0.2214	0.2107	0.2219	0.1979			
	Semotilus corporalis	AC	0.2555	0.2384	0.2385	0.2456	0.2456	0.2343	0.2409	0.2476	0.2426	0.2395	0.2602	0.2622	0.2562	0.2617	0.2308	0.2372	0.2274	0.2251	0.2281	0.2298	0.2250	0.2256	0.2273	0.2305	0.2247	0.2312	0.2144	0.1901		
Outgroups	*Cyprinus carpio*	AD	0.2640	0.2387	0.2375	0.2440	0.2440	0.2319	0.2295	0.2386	0.2304	0.2329	0.2639	0.2630	0.2629	0.2672	0.2291	0.2383	0.2353	0.2329	0.2308	0.2329	0.2248	0.2276	0.2259	0.2407	0.2274	0.2406	0.2338	0.2464	0.2649	
	Cobitis striata	AE	0.2949	0.2857	0.2827	0.2915	0.2915	0.2821	0.2833	0.2899	0.2789	0.2850	0.3005	0.2926	0.2956	0.3040	0.2730	0.2778	0.2709	0.2688	0.2725	0.2757	0.2666	0.2704	0.2700	0.2797	0.2684	0.2797	0.2792	0.2830	0.2939	0.2577

Note: A represents *Phoxinus grumi* ; B represents *Rhynchocypris czekanowskii* ; C represents *Rhynchocypris lagowskii* ; D represents *Rhynchocypris oxycephala* ; E represents *Rhynchocypris percnurus* ; F represents *Pseudaspius brandtii* ; G represents *Pseudaspius hakonensis* ; H represents *Pseudaspius leptocephalus* ; I represents *Pseudaspius nakamurai* ; J represents *Pseudaspius sachalinensis* ; K represents *Phoxinus bigerri* ; L represents *Phoxinus csikii* ; M represents *Phoxinus phoxinus* ; N represents *Phoxinus ujmonensis* ; O represents *Chrosomus erythrogaster* , P represents *Chrosomus tennesseensis* ; Q represents *Gila coerulea* ; R represents *Gila cypha* ; S represents *Gila elegans* ; T represents *Gila nigrescens* ; U represents *Ptychocheilus lucius* ; V represents *Ptychocheilus umpquae* ; W represents *Ptychocheilus oregonensis* ; X represents *Siphateles alvordensis* ; Y represents *Siphateles bicolor* ; Z represents *Siphateles boraxobius* ; AA represents *Couesius plumbeus* ; AB represents *Semotilus atromaculatus* ; AC represents *Semotilus corporalis* ; AD represents *Cyprinus carpio* ; AE represents *Cobitis striata* .
